# Individual bacterial cells can use spatial sensing of chemical gradients to direct chemotaxis on surfaces

**DOI:** 10.1038/s41564-024-01729-3

**Published:** 2024-09-02

**Authors:** James H. R. Wheeler, Kevin R. Foster, William M. Durham

**Affiliations:** 1 Department of Physics and Astronomy, University of Sheffield, Sheffield, UK; 2 Department of Biology, University of Oxford, Oxford, UK; 3 Department of Biochemistry, University of Oxford, Oxford, UK

**Keywords:** Cellular motility, Chemotaxis, Biofilms, Bacterial physiology, Cellular microbiology

## Abstract

Swimming bacteria navigate chemical gradients using temporal sensing to detect changes in concentration over time. Here we show that surface-attached bacteria use a fundamentally different mode of sensing during chemotaxis. We combined microfluidic experiments, massively parallel cell tracking and fluorescent reporters to study how *Pseudomonas aeruginosa* senses chemical gradients during pili-based ‘twitching’ chemotaxis on surfaces. Unlike swimming cells, we found that temporal changes in concentration did not induce motility changes in twitching cells. We then quantified the chemotactic behaviour of stationary cells by following changes in the sub-cellular localization of fluorescent proteins as cells are exposed to a gradient that alternates direction. These experiments revealed that *P. aeruginosa* cells can directly sense differences in concentration across the lengths of their bodies, even in the presence of strong temporal fluctuations. Our work thus overturns the widely held notion that bacterial cells are too small to directly sense chemical gradients in space.

## Abstract (web summary)

Microfluidic experiments reveal that surface-attached *Pseudomonas aeruginosa* cells directly sense differences in chemical concentration across the length of their cell bodies to guide pili-based chemotaxis.

## Main

**Fig. 1:**
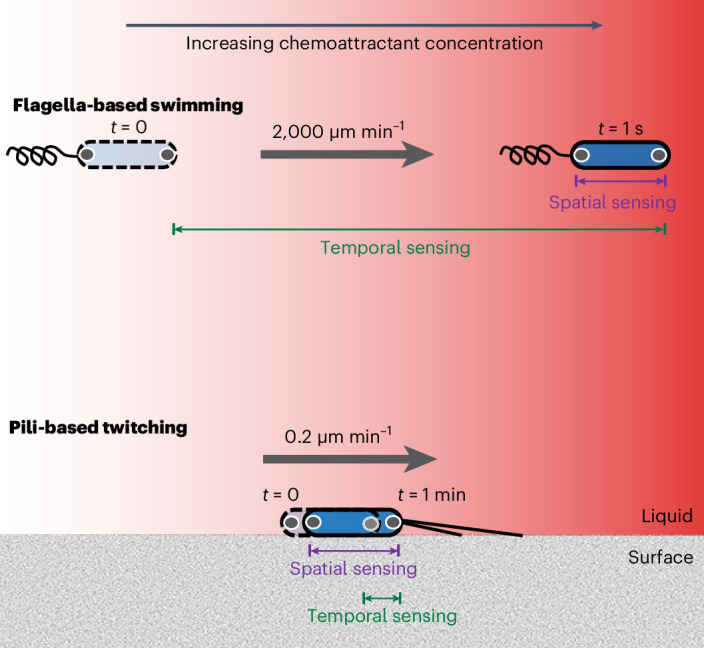
Swimming bacteria experience larger changes in concentration over time, whereas twitching bacteria experience larger changes in concentration over the lengths of their bodies. In principle, chemotaxing cells could either sense changes in chemoattractant concentration by moving from one location to another and comparing how the concentration changes over time (temporal sensing) or by directly comparing differences in concentration over the length of their bodies (spatial sensing). The rapid speed of swimming bacteria (for example, ref.^39^) means that over the course of their typical response time (on the order of 1 s), they would experience a larger change in concentration in time than space (denoted by the green and purple bars, respectively). The opposite is true for solitary surface-attached twitching bacteria, which move much more slowly (Extended Data Fig. 1c) and have response times on the order of 1 min^33^. Here chemoreceptor clusters are represented by the grey circles within the cell poles.

Cellular chemotaxis, the ability to sense chemical gradients and actively direct motility along them, plays a central role in many important processes including disease^1,2^, foraging^3,4^, sexual reproduction^5^ and multicellular development^6,7^. There are two distinct ways that cells can sense chemical gradients (Fig. 1). Cells using temporal sensing measure changes in chemical concentration over time as they travel along gradients. By contrast, cells using spatial sensing directly compare the concentration of a chemical at different positions along their cell body, independently from cell movement. The two sensing mechanisms are not necessarily mutually exclusive; in some complex signal transduction systems (for example, in certain eukaryotic cells that travel along surfaces using amoeboid movement), they can also be used in combination to guide chemotaxis^8^.

While eukaryotic cells are capable of both forms of sensing, the paradigm in the study of bacterial chemotaxis is one of temporal sensing. In particular, whenever the chemosensory systems of swimming bacteria have been characterized in detail, they have exclusively been found to use temporal sensing mechanisms to detect chemical gradients^9–15^, and these are particularly well understood in swimming *Escherichia coli*^16–20^. Temporal sensing allows fast-swimming bacteria to measure changes in concentration that occur over length scales equivalent to tens of cell body lengths (Fig. 1 and Supplementary Information), enabling them to better distinguish chemical gradients from stochastic noise. However, the advantage conferred by temporal sensing is predicted to scale with movement speed, and theoretical models suggest that spatial sensing could potentially confer increased sensitivity to bacteria-sized swimming cells in some parameter regimes^21,22^. Despite this, there is only one potential observation of spatial sensing in bacteria, which was suggested as an explanation for the U-shaped trajectories made by an uncultured bacterium collected from marine sediments that swims using flagella extending from each of its two poles^23^. However, these analyses were not definitive, and swimming bacteria are generally understood to use temporal sensing to guide chemotaxis^20,24–29^.

This focus on swimming cells contrasts with the fact that most bacteria live in surface-attached communities called biofilms^30–32^. Flagella are ineffective at driving motility in surface-attached cells^33–35^; instead they propel themselves using other forms of motility^36,37^. For instance, many surface-attached bacteria move via twitching motility, which is driven by the extension and retraction of type IV pili that function like molecular grappling hooks to pull cells across surfaces^38^. It was previously demonstrated that individual *Pseudomonas aeruginosa* cells can use twitching motility to navigate chemoattractant gradients^33^. Specifically, when exposed to a chemoattractant gradient that alternated direction, surface-attached cells were observed to rapidly reverse direction in response, typically before travelling a single micron. In contrast to swimming cells that reverse direction by switching the direction of flagellar rotation^39^, twitching cells reverse direction by switching pili activity to the opposite pole of their rod-shaped bodies^40,41^. However, it is not known how surface-attached *P. aeruginosa* cells resolve which of their poles is directed toward higher chemoattractant concentrations as they navigate chemical gradients.

A priori, there are good reasons to suspect that surface-attached *P. aeruginosa* cells might use a different type of gradient sensing compared to swimming cells (Fig. 1 and Supplementary Information). On average, solitary twitching cells migrate approximately four orders of magnitude more slowly than swimming cells^33,39^. Whereas swimming bacteria typically cover a distance equivalent to tens of body lengths within the characteristic time it takes for them to respond to chemoattractant gradients (~1 s; ref. ^42^), twitching *P. aeruginosa* cells typically only move less than one fifth of their cell body length in their characteristic response time (~1 min; ref. ^33^). Swimming cells would thus detect a larger change in concentration by sensing temporal changes as they move, whereas the opposite is true for twitching cells, which could measure a larger change in concentration across the length of their bodies (Fig. 1). While surface-attached bacteria are known to detect non-chemical stimuli, such as light and mechanical forces (both of which are intrinsically vectorial), over the lengths of their bodies^41,43^, we currently do not know whether they are also capable of sensing chemical concentration (which is a scalar) in analogous fashion. We therefore decided to investigate whether surface-attached *P. aeruginosa* cells, like eukaryotes, can detect chemical gradients across their cell bodies. To accomplish this, we used a series of microfluidic experiments to measure the response of individual solitary bacteria as they were exposed to different types of chemical stimulus.

## Results

### Twitching cells do not respond to temporal gradients

**Extended Data Fig. 1:**
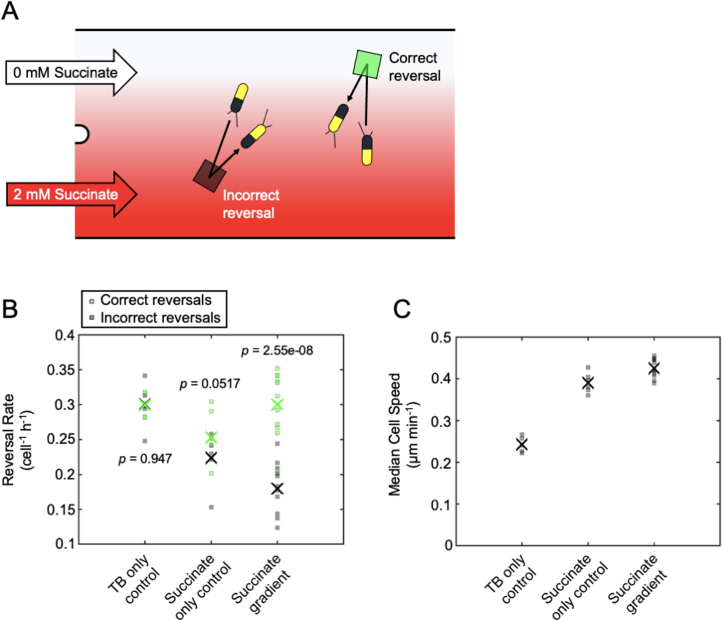
Surface-attached *P. aeruginosa* cells climb spatial succinate gradients by actively changing the rate at which they reverse direction. (**a**) A dual-inlet BioFlux microfluidic device was used to expose cells to a spatial gradient of succinate (*C*_MIN_ = 0 mM, *C*_MAX_ = 2 mM) with a characteristic length-scale of 100 µm, and an automated algorithm was used to detect when cells reversed their movement direction^33^. Reversals were classified as either ‘correct’ or ‘incorrect’. Correct reversals (green square) occur in cells that were initially moving away from the source of succinate, whilst incorrect reversals (black square) occur in cells that were initially moving towards the source of succinate. (**b**) The rate of correct reversals is significantly greater than that of incorrect reversals, which drives chemotaxis towards succinate. Crosses (‘X’) mark the mean of five separate TB-only control experiments, six separate succinate-only control experiments, or eleven separate succinate gradient experiments. In TB-only experiments, TB is passed through both inlets at the same time, whilst in succinate-only experiments, media containing succinate is passed through both inlets at the same time. Controls were processed in the same way as for succinate gradients, but since no gradient was actually present, the ‘correct’ and ‘incorrect’ rates shown are arbitrary. *p*-values were obtained from two-sided paired *t*-tests of the null hypothesis that the measured incorrect and correct reversal rates come from the same distribution. (**c**) Cell speed is significantly higher in the presence of succinate gradients compared to both control experiments and is significantly higher in succinate-only controls compared to TB-only controls (one-way ANOVA, *p* = 2.22 × 10^−11^). The analysis in panels B and C only includes trajectory time-points that occurred before 5 h, after which the cell density becomes too high to reliably track cells in these experiments. The succinate gradient datasets additionally only include trajectory time-points above a minimum gradient strength of 0.0006 mM µm^−1^ to ensure that cells within regions of the device where there is a very small spatial gradient were not included in our analysis. Source data provided as a Source Data file. Source data

While one can argue how spatial sensing might benefit twitching *P. aeruginosa* cells (Fig. 1 and Supplementary Information), it is well documented that bacteria use temporal mechanisms when swimming. We therefore began by testing whether temporal changes in chemoattractant concentration could explain the directed motility of *P. aeruginosa* on surfaces. The experiments that documented pili-based chemotaxis used a dual-flow microfluidic device where molecular diffusion mixes two streams of fluid with different chemoattractant concentrations as they flow down the length of the device (Extended Data Fig. 1 and ref. ^33^). In these assays, cells undergoing chemotaxis simultaneously experience a spatial gradient over the length of their bodies as well as temporal changes in chemoattractant concentration as they move along the gradient. This makes it difficult to ascertain whether cells are responding to either spatial or temporal stimuli.

**Fig. 2:**
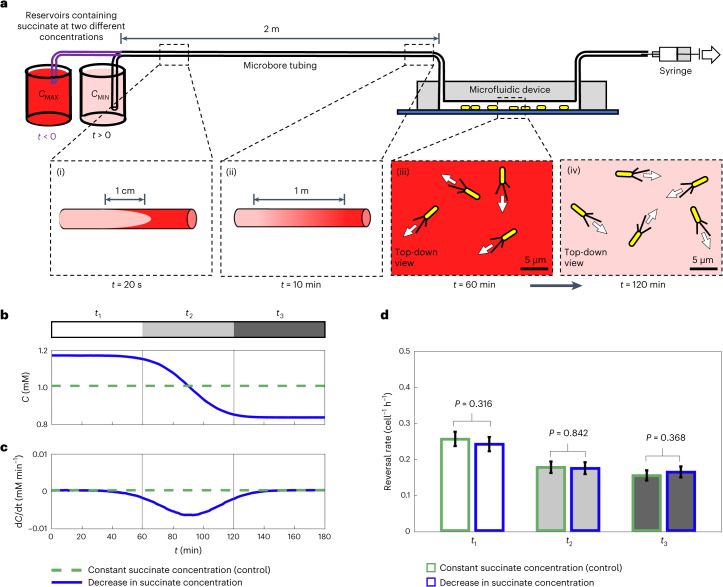
Temporal changes in concentration do not induce a chemotactic response in surface-attached *P. aeruginosa.* **a**, We used Taylor–Aris dispersion to generate concentration gradients along a 2 m long tube, which then flowed past surface-attached cells in microfluidic devices. We filled the system with media containing succinate (*C*_MAX_ = 1.16 mM). At *t* = 0, media containing a lower succinate concentration (*C*_MIN_ = 0.84 mM) was pulled through the system. As fluid moves fastest along a tube’s centreline, a plug of lower concentration fluid forms (panel i) but is rapidly mixed across the tube width via molecular diffusion (panel ii). The fluid interface forms a longitudinal gradient with an ~1.6 m length scale such that surface-attached cells within the device experience smooth temporal decreases in concentration (panel iii to panel iv). **b**,**c**, Using dye, we quantify succinate concentration (**b**) and temporal concentration gradient over time (**c**) (blue lines; dashed green lines show a control with 1 mM succinate throughout). Cells experience approximately the same mean temporal concentration gradient that cells experience in dual-inlet chemotaxis experiments (Extended Data Fig. 1 and ref. ^33^), but with ~16,000-fold smaller spatial gradients. **d**, In the 1 h period before the succinate gradient entered the device (interval *t*_1_), cell reversal rates were statistically indistinguishable between experiment (white bar, blue outline) and control (white bar, green outline; one-sided exact Poisson test (Methods) yielded *P* = 0.316). Similarly, reversal rates in the presence of a temporal succinate gradient (interval *t*_2_; light grey bar, blue outline) and in the 1 h period after the gradient had cleared the microfluidic device (interval *t*_3_; dark grey bar, blue outline) were statistically indistinguishable from the reversal rates in the control (*P* = 0.842 and *P* = 0.368). The number of reversals observed was *n*_r_ = 1,496 and 1,391 across *n*_t_ = 468,596 and 439,632 trajectory points in the control and experimental conditions, respectively. Error bars show 95% confidence intervals about the mean reversal rates assuming that reversals follow a Poisson distribution (Methods). Data shown here are representative of two bio-replicates (Extended Data Fig. 5). Source data provided as a Source data file. Source data

To directly test whether twitching cells use temporal signals to guide chemotaxis, we developed a custom microfluidic set-up that uses Taylor–Aris dispersion^44,45^ to generate a concentration gradient of succinate (a known chemoattractant and preferred carbon source of *P. aeruginosa*; Extended Data Fig. 1 and ref. ^33^) that flows past cells. Importantly, our custom microfluidic set-up exposes all cells to an approximately equal temporal stimulus, independent of their movement speed or direction (Fig. 2 and Methods). Twitching cells in dual-flow microfluidic experiments bias their motility towards succinate by both increasing and decreasing their reversal frequency when moving away from or towards chemoattractants, respectively, compared to a control that contains a uniform concentration of succinate (Extended Data Fig. 1). Therefore, if cells indeed used temporal measurements to guide chemotaxis, we would expect that a temporal decrease in succinate concentration would cause the cells in our Taylor–Aris dispersion experiments to reverse more frequently, and vice versa.

We designed our Taylor–Aris dispersion experiments to expose cells to the same average chemical temporal stimuli that cells experienced in the dual-flow experiments where chemotaxis was originally demonstrated. This correspondence was accomplished by matching both the concentrations (*C*) and mean temporal concentration gradients (d*C*/d*t*) that cells experience in those experiments (Methods). Importantly, in our Taylor–Aris dispersion experiments, the chemoattractant gradient forms over the length of a 2-m-long tube leading to the microfluidic device (Fig. 2a), such that the chemical gradient measures approximately 1.6 m in length by the time it reaches the cells. By contrast, in dual-flow experiments, the gradient instead forms across the width of the microfluidic device and has a characteristic length scale of 100 µm. Therefore, the cells in our Taylor–Aris dispersion experiments experience approximately a 16,000-fold smaller gradient across the length of their bodies (that is, d*C*/d*x*) compared to the dual-inlet experiments, while experiencing approximately the same mean temporal stimuli (d*C*/d*t*).

**Extended Data Fig. 2:**
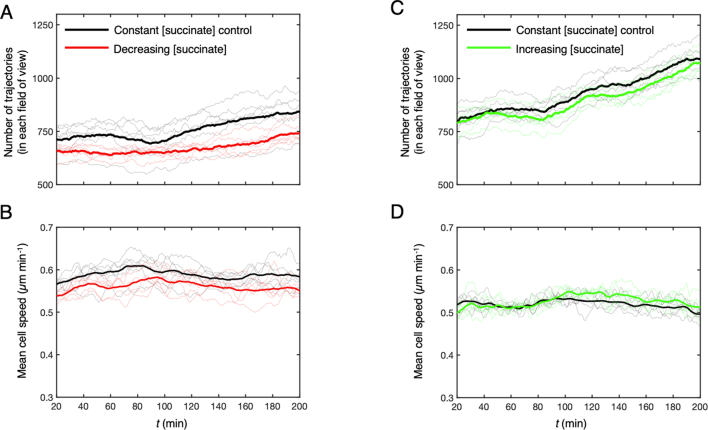
The effect of succinate on cells in our Taylor-Aris dispersion experiments. (**a**) Thin lines show the number of cell trajectories that were imaged in each of the six simultaneously imaged fields of view that were used in our Taylor-Aris dispersion experiments, whilst the thick lines show the mean. We observed that the number of cells increased gradually over the course of our approximately 3 h long experiments, regardless of whether cells were exposed to a decrease in succinate concentration over time (red lines) or to a constant concentration of succinate *C* = 1 mM in control experiments (black lines). (**b**) Cell speed remained approximately constant both in controls (black lines) and in cells exposed to decreasing succinate concentration (red lines). (**c**, **d**) Similar trends were observed for cells exposed to an increase (green lines) in succinate concentration over time when compared to their respective controls (black lines). The data shown here is representative of both bio-replicates. Source data provided as a Source Data file. Source data

**Extended Data Fig. 3:**
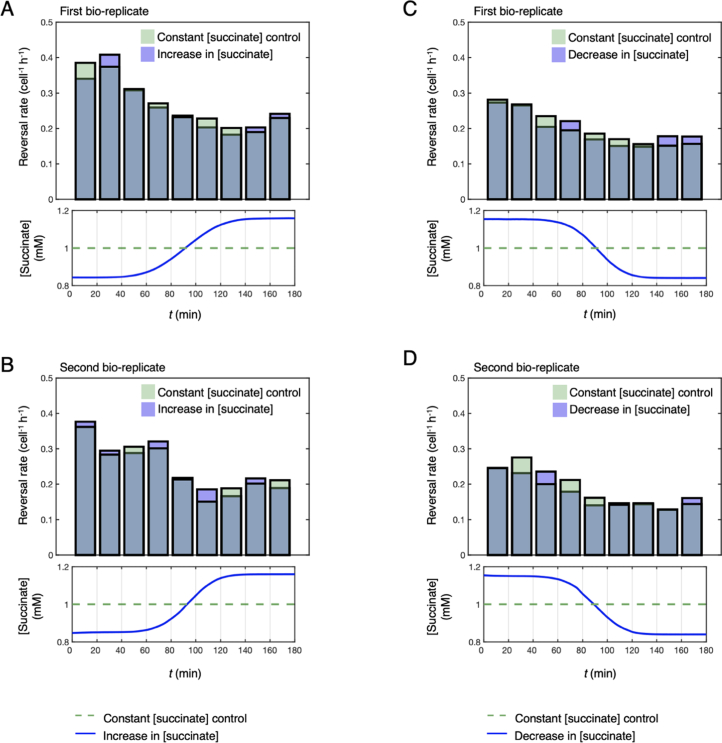
Cell reversal rate decreases over time in our Taylor-Aris dispersion experiments and in their respective controls. (**a**) In experiments that exposed cells to a temporal increase in succinate concentration (blue bars), cell reversal rate decreased over the time course of the experiments. A similar decrease was observed in the corresponding controls (green bars) where cells were exposed to a constant succinate concentration *C* = 1 mM. Similar trends were observed in a second bio-replicate of this experiment (**b**) and in two bio-replicates where cells were exposed to a temporal decrease in succinate concentration (**c**, **d**). Source data provided as a Source Data file. Source data

**Extended Data Fig. 4:**
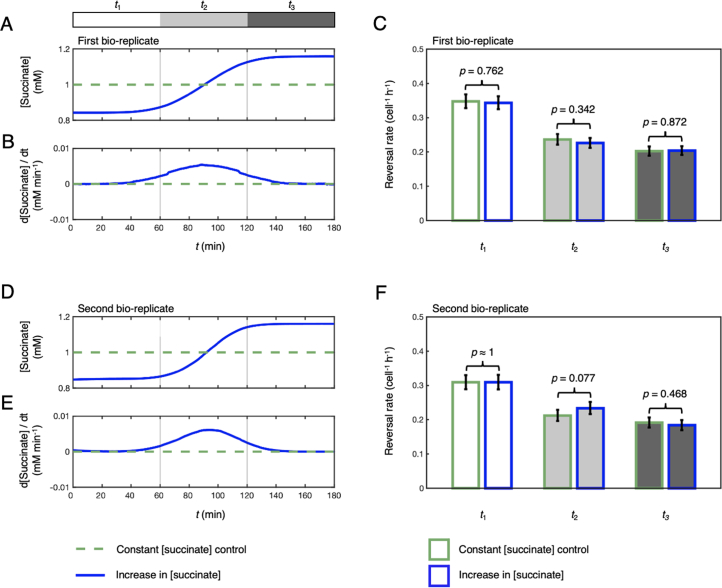
A temporal increase in succinate concentration does not induce a chemotactic response in surface-attached *P. aeruginosa.* (**a**, **b**) Using the approach outlined in Fig. 2, cells were exposed to temporal increases in succinate concentration (*C*_MIN_ = 0.84 mM, *C*_MAX_ = 1.16 mM; blue line). This generates mean temporal concentration gradients approximating the gradient experienced by cells moving towards increasing succinate concentrations in the dual-inlet chemotaxis experiments (Extended Data Fig. 1), but with 16,000-fold smaller spatial gradients. If cells can sense these temporal stimuli, the temporal increase in succinate concentration would be predicted to suppress reversals. (**c**) In the 1 h period before the succinate gradient entered the microfluidic device (interval *t*_1_) cell reversal rates were statistically indistinguishable between experiment (white bar, blue outline) and control (white bar, green outline; one-sided exact Poisson test (**Methods**) yielded *p* = 0. 762). Similarly, the reversal rates in the presence of a temporal succinate gradient (interval *t*_2_; light grey bar, blue outline) and in the 1 h period after the gradient had cleared the microfluidic device (interval *t*_3_; dark grey bar, blue outline) were statistically indistinguishable from the reversal rates during the same time periods in the control (*p* = 0.342 and *p* = 0.872). The number of reversals observed was *n*_r_ = 2709 and 2980 across *n*_t_ = 636,364 and 709,607 trajectory points in the control and experimental conditions respectively. Error bars show 95% confidence intervals about the estimated reversal rates assuming that reversals follow a Poisson distribution (**Methods**). (**d**, **e**, **f**) A second bio-replicate confirmed that when comparing between experiment and control, reversal rates were indistinguishable during time periods *t*_1_ (white bars, *p* ≈ 1), *t*_2_ (light gray bars, *p* = 0.077) and *t*_*3*_ (dark gray bars, *p* = 0.468). p-values were obtained from a one-sided exact Poisson test (**Methods**); *n*_r_ = 2101 and 2034 across *n*_t_ = 536,892 and 504,264 trajectory points in the control and experimental conditions respectively. Error bars show 95% confidence intervals about the mean reversal rates, assuming that reversals follow a Poisson distribution (**Methods**). Source data provided as a Source Data file. Source data

**Extended Data Fig. 5:**
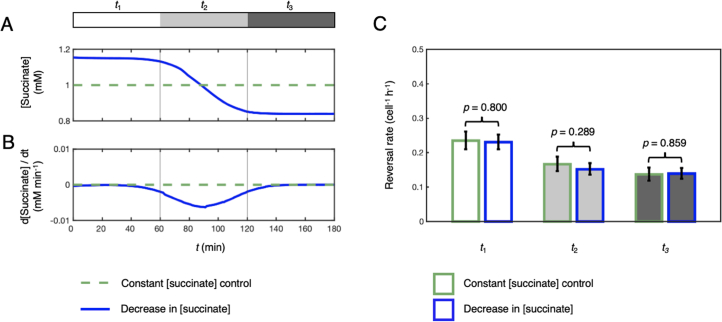
A temporal decrease in succinate concentration does not induce a chemotactic response in surface-attached *P. aeruginosa.* (**a**, **b**) Data shown come from a biological repeat of the experiment outlined in Fig. 2, where cells were either exposed to a temporal decrease in succinate concentration over time (blue lines) or to a control with a constant succinate concentration *C* = 1 mM (dashed green lines). (**c**) Using automated reversal detection, we first confirmed that the reversal rate in the 1 h period before the succinate gradient entered the microfluidic device (time interval *t*_1_; white bar, blue outline), was statistically indistinguishable from the reversal rate during the same time period in a simultaneous control experiment where a constant concentration of succinate was maintained throughout (white bar, green outline). Specifically, a one-sided exact Poisson test (**Methods**) did not reject the null hypothesis that these two reversal rate measurements come from the same Poisson distribution, *p* = 0.800. Similarly, the reversal rates in the presence of a temporal succinate gradient (time interval *t*_2_; light grey bar, blue outline) and in the 1 h period after the gradient had cleared the microfluidic device (time interval *t*_3_; dark grey bar, blue outline) were statistically indistinguishable from the reversal rates during the same time periods in the control (*p* = 0.289 and *p* = 0.859). The total number of reversals observed in our six simultaneously imaged fields of view was *n*_r_ = 772 and 1072 across a total of *n*_t_ = 259,301 and 370,801 trajectory points in the control and experimental conditions respectively. Error bars show 95% confidence intervals about the mean reversal rates assuming that reversals follow a Poisson distribution (**Methods**). Source data provided as a Source Data file. Source data

We used massively parallel cell tracking and automated reversal detection^33^ to simultaneously quantify the movement of thousands of cells attached to the surface of a microfluidic device (Extended Data Fig. 2). In addition to exposing cells to temporal gradients of succinate, we ran a control experiment in an adjacent microfluidic channel on the same microscope where cells were exposed to a constant succinate concentration over time. This control allows us to distinguish any potential changes in cell motility induced by the temporal succinate gradient from other, more general changes in cell motility over time. For example, an increase in the amount of exopolysaccharides present on the surface^46^ or physiological adaptation of cells to the surface (mediated, for example, by surface sensing and second messengers such as cyclic adenosine monophosphate^47–49^) could change cell motility over time (Extended Data Figs. 2 and 3). To control for such effects, we established a baseline by analysing cell motility in the 1 h period that preceded the succinate gradient entering the microfluidic device (white region labelled *t*_1_ in Fig. 2b,c) and compared it to that measured over the same time period in the control. As reversals are relatively rare events^33^, we imaged six fields of view in each channel, which allowed us to track approximately 10^4^ cells simultaneously (Extended Data Fig. 2). We found that the baseline reversal rate before the gradient entered the microfluidic channel (white region labelled *t*_1_ in Fig. 2b) was statistically indistinguishable when compared with the reversal rate observed in the control over the same time period (Fig. 2d and Extended Data Figs. 4 and 5). This strong correspondence thus indicates that we can directly compare the cellular reversal rates in the two channels at later time points to assess whether a temporal gradient in concentration causes cells to alter their reversal rate.

We next calculated the reversal rate of cells as they experienced a temporal decrease or increase in succinate concentration (light grey region labelled *t*_2_ in Fig. 2b,c) and compared it to that measured over the same time period in the constant succinate concentration control. Regardless of whether cells were exposed to a temporal increase or decrease in succinate concentration, cell reversal rates in time period *t*_2_ were statistically indistinguishable when compared between experimental and control conditions (Fig. 2d and Extended Data Figs. 4 and 5). Finally, we measured reversal rates in the 1 h time period after the temporal gradient had cleared the microfluidic device to confirm that the gradients did not have a latent effect on cell reversal rates (dark grey region labelled *t*_3_ in Fig. 2b,c). Once again, cell reversal rates in time period *t*_3_ were statistically indistinguishable when comparing between the control and experimental conditions (Fig. 2d and Extended Data Figs. 4 and 5). Taken together, our results thus strongly suggest that cells do not alter their reversal rate in response to temporal succinate gradients. While it is known that twitching cells generate chemotaxis by actively modulating their reversal frequency in response to the direction that they are travelling along a chemoattractant gradient (Extended Data Fig. 1; ref. ^33^), the absence of a response in our Taylor–Aris dispersion experiments suggests that *P. aeruginosa* cells do not use the mean temporal changes in concentration they experience to guide pili-based chemotaxis.

**Extended Data Fig. 6:**
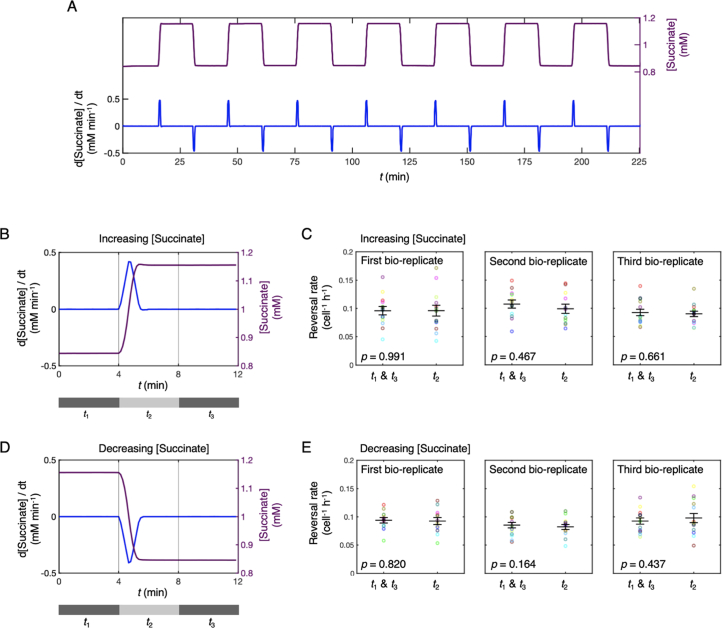
Steep, rapid temporal chemoattractant gradients do not cause surface-attached *P. aeruginosa* cells to change their reversal rate. (**a**) Twitching is characteristically jerky and cells could have evolved to detect the large but ephemeral temporal changes in chemoattractant concentration caused by these intermittent displacements. To test this hypothesis, we used a microfluidic setup that exposed surface-attached cells to rapid temporal changes in succinate concentration (see **Methods**). We used dye to quantify temporal changes in succinate concentration (purple line) and the temporal succinate gradients (blue line) that cells experienced in these experiments. In this experiment, cells are repeatedly exposed to both increases and decreases in succinate concentration. (**b**) To analyse cells’ response to these different stimuli, we first split reversal data around each increase in succinate concentration into three time-bins *t*_1_, *t*_2_, and *t*_3_ corresponding to the 4 min intervals before, during and after the temporal gradient. (**c**) Reversal rates were pooled across time windows *t*_1_ and *t*_3_, corresponding to time periods without any succinate concentration gradients, and compared to the reversal rates during the temporal increases in succinate concentration, time-bin *t*_2_. The mean reversal rate measured during the temporal increase in succinate concentration (large black ‘-’ marker) was statistically indistinguishable from that when the succinate concentration was constant (a two-tailed, paired *t*-test of the null hypothesis of no difference in reversal rates yielded *p* = 0.991, 0.467 and 0.661 for three independent bio-replicates). Mean reversal rates were averaged across six subsequent increases in succinate concentration (see (A)) each imaged across two independent fields of view (the 12 circular markers are colour-coded to show pairs of data recorded in each of the 12 fields of view, see **Methods**). (**d**, **e**) Similar results were obtained when comparing reversal rates between the presence (*t*_2_) and absence (*t*_1_ and *t*_3_) of temporal decreases in succinate concentration (a two-tailed, paired *t*-test of the null hypothesis of no difference in reversal rates yielded *p* = 0.820, 0.164 and 0.437 for three independent bio-replicates). Error bars show mean reversal rates plus and minus standard error. Source data provided as a Source Data file. Source data

However, we decided to explore another possible basis for temporal sensing. While the Taylor–Aris dispersion experiments simulated the long-term, average temporal changes in concentration experienced by cells in experiments where chemotaxis was observed, on shorter timescales, twitching cells routinely undergo much more rapid movement caused by the stochastic release of individual pili^38,50^. These rapid movements can momentarily transport cells at speeds that are approximately 20-fold larger than their movement speeds during their more regular, slower form of movement, and thus they could expose cells to larger temporal stimuli (Supplementary Information). This is because the magnitude of the temporal gradient a cell experiences scales with cell velocity, *V*_C_, relative to a chemical gradient like d*C*/d*t* = *V*_C_ d*C*/d*x*. Therefore, to measure the response of twitching cells to more rapid changes in succinate concentration, we used a programmable microfluidic system that smoothly switches between two different concentrations of succinate over a period of 1.5 min, yielding temporal gradients, d*C*/d*t*, that are approximately 40-fold larger than the experiments shown in Fig. 2c (Methods). Given the short timescale of these temporal gradients, we alternated between two different succinate concentrations >12 times over the course of each experiment, allowing us to expose the same cells to both positive and negative temporal concentration gradients and analyse data across them separately. While these temporal gradients were much sharper than those in the Taylor–Aris dispersion experiments, we again found that temporal stimuli did not generate any detectable changes in cell reversal rates (Extended Data Fig. 6). Taken together, these first experiments strongly suggest that surface-attached *P. aeruginosa* do not use temporal stimuli to determine whether they are moving up or down a chemical gradient.

### Quantifying chemotactic behaviour in stationary cells

Our first experiments indicated that twitching chemotaxis is not driven by temporal sensing, suggesting instead that *P. aeruginosa* cells might directly sense differences in concentration across the length of their bodies. However, to evaluate this possibility, we needed to find a way to experimentally decouple the spatial and temporal information that cells experience. The challenge is that a cell moving through a steady spatial gradient of chemoattractant will experience differences in concentration along the length of its body, while simultaneously experiencing changes in concentration over time as it moves relative to the gradient. To decouple these two different stimuli from one another, we decided to study the behaviour of stationary cells, which typically make up a relatively small percentage of cells within our microfluidic assays (approximately 5–10%). The question then was how does one characterize chemotactic behaviour in cells that are not moving?

Here, we initially found inspiration in the studies of *Myxococcus xanthus*, which can also move via twitching motility^51^. Reversals occur 40 times more frequently in *M. xanthus* and are accompanied by changes in the sub-cellular localization of two motor proteins, PilB and PilT, which are responsible for pili extension and retraction, respectively^52–54^. In twitching *M. xanthus* cells, PilB localizes to the front pole of a moving cell (the ‘leading pole’), whereas PilT localizes predominantly to the rear pole (the ‘trailing pole’). The two motor proteins then switch between the two poles of *M. xanthus* cells during reversals. If these motor proteins show similar patterns of localization in twitching *P. aeruginosa* cells, we could potentially use fluorescent fusions to quantify reversals in cell polarity, even in cells that are temporarily stationary.

**Fig. 3:**
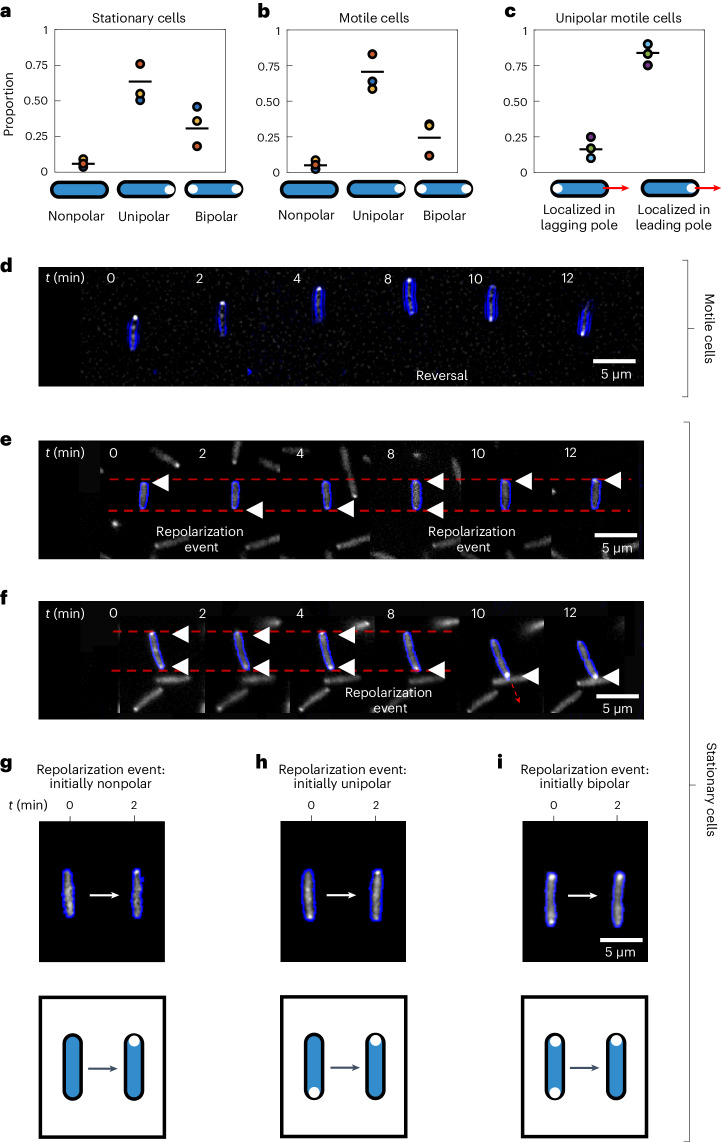
PilT–YFP localizes to the leading pole of motile cells and can dynamically re-localize within the bodies of both motile and stationary cells, providing a means to infer chemotactic behaviour. **a**,**b**, In the majority of both stationary (**a**) and motile (**b**) cells, the PilT–YFP fusion protein localizes to one of the two cell poles (unipolar). A smaller proportion of cells have PilT–YFP localizations in both poles (bipolar) or lack appreciable localizations altogether (nonpolar). Black lines show the mean of three bio-replicates that were each conducted on different days, represented here with a different coloured circle. The data from each bio-replicate contained over *n* = 1,000 trajectories. **c**, If we consider only those motile cells that have a unipolar PilT–YFP localization, we find that PilT–YFP is significantly more likely to localize to a cell’s leading pole (mean proportion = 0.84; a two-sided binomial test of proportions rejects the null hypothesis of equal proportions with *P* < 1 × 10^−10^ for each bio-replicate, assuming that data from each cell at each time point are independent measurements). **d**, A time series of a motile twitching cell (cell outline shown in blue) undergoing a reversal at *t* = 8 min. PilT–YFP (shown in white) localizes to the leading pole, so that it swaps from one pole to the other when the cell reverses direction. **e**, A time series of a stationary cell reveals that PilT–YFP can swap between a cell’s two poles over time, an event we call a ‘repolarization event’. Localizations of PilT–YFP are marked with white triangles. **f**, A cell that is initially stationary has PilT–YFP localized to both of its poles, but subsequently PilT–YFP accumulates within its bottom pole shortly before the cell initiates movement in the downward direction. Faint dashed red lines in **e** and **f** mark the position of the two cell poles in the first image of the time series. **g**–**i**, Repolarization events can occur in cells that are initially nonpolar (**g**), unipolar (**h**) or bipolar (**i**). Cells shown are representative of three bio-replicates. Source data provided as a source data file. Source data

**Extended Data Fig. 7:**
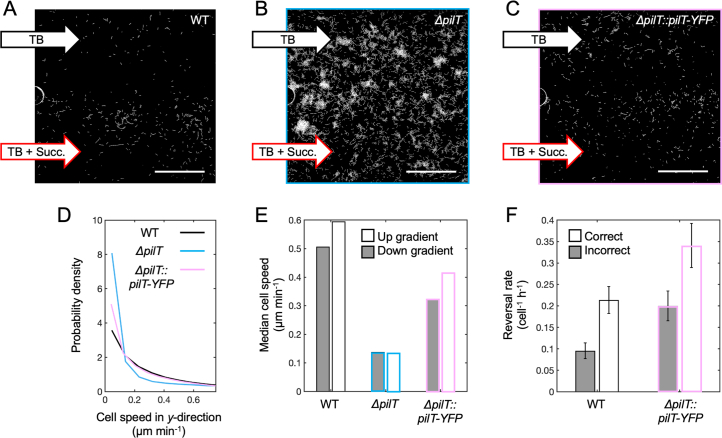
The PilT-YFP fusion protein complements motility and chemotaxis phenotypes of a *ΔpilT* mutant. (**a**) Surface-attached *P. aeruginosa* cells within dual-inlet microfluidic devices (scale bars = 100 µm) were exposed to succinate gradients by flowing TB through one inlet and TB media supplemented with succinate (*C*_MAX_ = 2 mM) through the other. WT cells (white) undergo chemotaxis and accumulate at high succinate concentrations (*t* =10 h). (**b**) *P. aeruginosa* cells lacking *pilT* (*ΔpilT*) have impaired twitching motility^55^ and distribute equally across the device (*t* = 10 h). (**c**) Our PilT-YFP translational fusion restores motility and chemotaxis when expressed in the *ΔpilT* strain (*ΔpilT*::*pilT-YFP*, *t* = 10 h). Images representative of two bio-replicates. (**d**) A probability density function of cell speed (first 300 min, when cells exhibit highest motility) confirms that the *ΔpilT* strain (blue line) has impaired twitching motility, which is restored by our PilT-YFP translational fusion (pink line). WT cell speeds (black line) are shown for reference. Cell speed was analysed in the *y*-dimension as cells are pushed slightly by fluid flow in the *x*-direction shown from left-to-right in panels A-C. (**e**) WT cells move up succinate gradients (white bars) faster than they move down them (grey bars), a trend also observed in our *ΔpilT*::*pilT-YFP* strain (pink bars). However, this trend is lacking in a *ΔpilT* mutant, which has greatly reduced overall cell speeds. All bars show median cell speeds. (**f**) WT and our *ΔpilT*::*pilT-YFP* strain deploy ‘correct’ reversals (white bars) more frequently than ‘incorrect’ reversals (grey bars, see Extended Data Fig. 1; a one-sided exact Poisson test (**Methods**) yields *p* = 0.0005 and *p* = 0.033 across a total of *n* = 89 and *n* = 156 reversals). We could not measure reversal rates for our *ΔpilT* strain because of its general lack of motility. Error bars show 95% confidence intervals centred on the mean reversal rates, assuming that reversals follow a Poisson distribution (**Methods**). Data shown is representative of two bio-replicates. Here, tryptone broth is ‘TB’ and succinate is ‘Succ’. Source data provided as a Source Data file. Source data

To visualize the retraction motor PilT in cells undergoing reversals, we fused PilT to yellow fluorescent protein (YFP) and expressed it in a *P. aeruginosa* strain lacking a functional native copy of PilT (*ΔpilT*::*pilT-yfp*; Methods and Table 1). This fusion protein complemented the motility of the *ΔpilT* strain (Extended Data Fig. 7), a mutant lacking the first portion of the gene’s coding region (Methods; ref. ^55^). We find that our PilT–YFP fusion protein localizes predominantly to the leading cell pole in twitching *P. aeruginosa* cells (Fig. 3b,c), which is consistent with two recent studies^41,56^, and implies that reversals in cell movement direction will be associated with a re-localization of PilT–YFP to a cell’s new leading pole (for example, Fig. 3d). Given that PilT instead localizes to the trailing pole in twitching *M. xanthus* cells, this implies that different molecular mechanisms are used to generate reversals in these two species.

In stationary cells, PilT–YFP can also localize to neither (‘nonpolar’), to one (‘unipolar’) or to both cell poles simultaneously (‘bipolar’; Fig. 3a). Crucially, we found that the localization of PilT–YFP remains dynamic in the stationary cells in our microfluidic assays, with new localizations forming and old localizations dissipating over time (Fig. 3e,f). These findings indicate that changes in the sub-cellular localization of PilT–YFP can be used to distinguish between the leading and lagging pole before a cell starts to move. Specifically, this fusion allows us to detect ‘repolarization events’ in stationary cells, which occur when PilT–YFP redistributes within the cell (Fig. 3g–i), and quantify how they are elicited by different types of chemical gradients. Tracking changes in the sub-cellular localization of PilT–YFP therefore allows us to analyse the chemotactic behaviour of stationary cells.

### Spatial sensing guides twitching chemotaxis

**Fig. 4:**
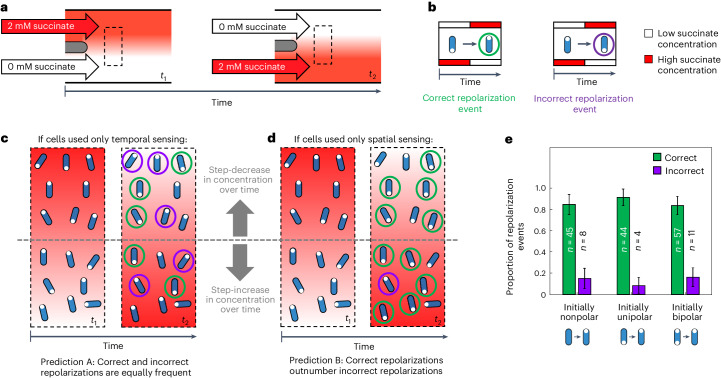
Repolarization events in stationary cells exposed to an alternating succinate gradient preferentially re-localize PilT–YFP to the cell pole experiencing larger succinate concentrations, indicating that they are capable of spatial sensing. **a**, We used a dual-flow microfluidic device to expose cells to a spatial gradient of succinate that alternates direction^33^. The dashed black box indicates the region downstream of the two inlets, where we imaged cells. **b**, In response to this alternating spatial gradient, stationary cells (blue) expressing PilT–YFP (white circles) can undergo either correct or incorrect repolarization events. **c**,**d**, The relative proportion of correct and incorrect repolarization events in this experiment can be used to determine whether cells use temporal (**c**) or spatial (**d**) sensing. **c**, Stationary cells using only temporal sensing could garner no information about a gradient’s spatial orientation and would therefore be equally likely to generate correct and incorrect repolarization events (prediction A). **d**, By contrast, stationary cells capable of spatial sensing could directly sense the gradient’s spatial orientation, allowing them to deploy correct repolarization events at a greater frequency than incorrect repolarization events (prediction B). **e**, Quantifying the behaviour of *n* = 171 stationary cells undergoing repolarization events within our alternating gradient experiments (see Supplementary Videos 1–16 and Supplementary Table 1) revealed that correct repolarization events occurred approximately 6 times more frequently than incorrect repolarization events, regardless of whether PilT–YFP localization was initially nonpolar, unipolar or bipolar (Fig. 3g–i). An exact two-tailed binomial test rejected the null hypothesis that correct and incorrect repolarization events were equally abundant with *P* = 2.37 × 10^−7^, 1.51 × 10^−9^ and 1.28 × 10^−8^ for nonpolar, unipolar and bipolar repolarization events, respectively. This is consistent with prediction B, indicating that cells are capable of directly sensing differences in concentration over the length of their bodies. Error bars show 95% confidence intervals about the proportion estimates. Source data provided as a Source data file. Source data

To test for spatial sensing, we used a custom Y-shaped microfluidic device^33^ to expose our *P. aeruginosa* (*ΔpilT*::*pilT-yfp*) cells to a spatial gradient of succinate that alternates in direction (Fig. 4a). We then followed the distribution of PilT–YFP within a total of >1,000 stationary cells and recorded whether or not these stationary cells underwent repolarization events when they were exposed to a succinate gradient that alternated direction approximately every 45–60 min (Methods). Stationary cells that underwent repolarization events can be separated into two different categories: ‘correct’ repolarization events in which cells re-localize PilT–YFP in the pole experiencing higher succinate concentrations and ‘incorrect’ repolarization events, where PilT–YFP is re-localized in the pole experiencing lower succinate concentrations (Fig. 4b).

The relative frequency of correct and incorrect repolarization events in stationary cells allows us to directly test whether cells respond to temporal or spatial stimuli. As stationary cells do not move appreciably relative to the gradient, the temporal stimuli they experience do not encode information that could allow them to determine the orientation of the chemical gradient. Instead, on one side of the device stationary cells simply experience an increase in concentration over time, whereas on the other side, they experience a decrease in concentration over time (Fig. 4c,d). Therefore, temporal sensing and spatial sensing lead to two different, and easily distinguishable, predictions in these experiments. If stationary cells used temporal sensing, repolarization events would be independent of the gradient’s orientation, so one would expect that correct and incorrect repolarization events would both occur randomly and, therefore, at approximately the same rate (‘prediction A’; Fig. 4c). By contrast, if stationary cells can make spatial measurements, we expect that correct repolarization events will occur more often than incorrect repolarization events. This is because cells that sense the direction of the chemical gradient by directly measuring it across their bodies would be able to correctly ascertain the gradient’s spatial orientation (‘prediction B’; Fig. 4d).

**Fig. 5:**
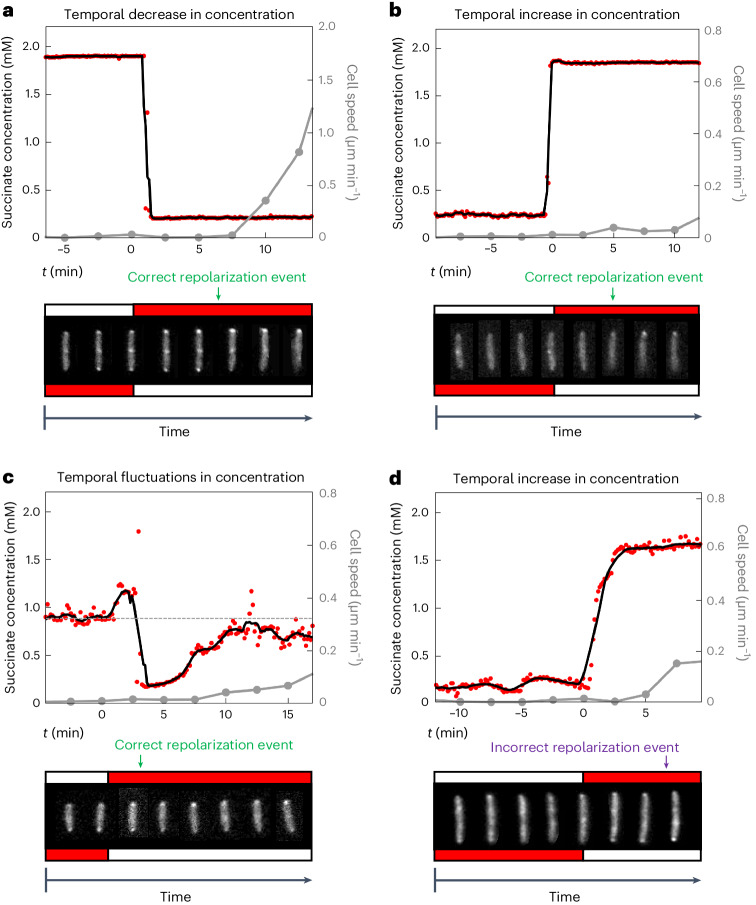
Stationary cells can sense changes in the orientation of a chemoattractant gradient, despite large temporal fluctuations in concentration. We simultaneously quantified the succinate concentration that a cell experienced over time (red circles; black line shows moving average), cell speed (grey line) and PilT–YFP localization, as cells were exposed to a succinate gradient that alternates direction. Grey circles indicate time points at which cell images are shown (at 2.5 min intervals). To guide the eye, cell images have been repositioned so that they are vertically oriented and their centroid remains at a fixed position. **a**, This cell experiences a sharp temporal decrease in succinate concentration when the gradient changes direction. PilT–YFP re-localizes to the cell pole that is now exposed to higher chemoattractant concentrations (a correct repolarization event), and the cell later moves off in the direction of its new leading pole. PilT–YFP is shown in the bottom inset, with red and white boxes indicating high and low succinate concentrations, respectively. **b**, This cell experiences a sharp increase in succinate concentration over time and also undergoes a correct repolarization event. While PilT–YFP is initially nonpolar, it subsequently re-localizes exclusively to the cell pole positioned in higher succinate concentrations. **c**, This cell was positioned close to the centreline of the succinate gradient such that when the gradient alternated direction, it experienced noisy fluctuations in succinate concentration, including both increases and decreases in concentration. Despite this, the cell also underwent a correct repolarization event—PilT–YFP was initially localized to both poles (with no observable directional polarity) and subsequently re-localized exclusively to the cell pole positioned in higher succinate concentrations. **d**, Although less frequent, cells also underwent incorrect repolarization events. Here a cell experiencing an increase in succinate concentration over time re-localizes PilT–YFP to the cell pole positioned in lower succinate concentrations and subsequently moves in that direction. While these four repolarization events are representative, Supplementary Videos 1–16 show every repolarization event that we observed, with a description of how each was classified in Supplementary Table 1. Source data provided as a source data file. Source data

**Extended Data Fig. 8:**
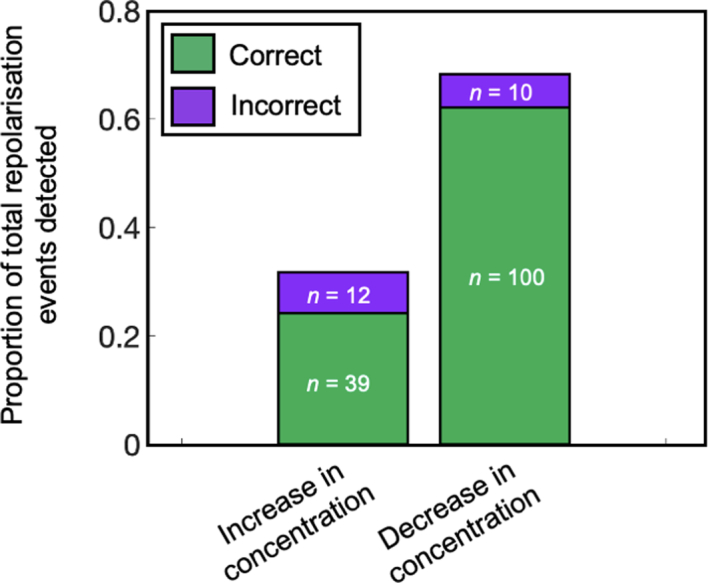
Stationary cells are more likely to undergo repolarisation events when they have recently experienced a rapid decrease in chemoattractant concentration. In the alternating gradient experiments (Figs. 4 and 5), cells are exposed to large temporal changes in succinate concentration. We observed a larger number of repolarisation events in cells that experienced a temporal decrease in succinate concentration (68% of the total) than those that experienced a temporal increase (32% of the total). Specifically, we rejected the null hypothesis that the proportion of repolarisation events that occurred in cells experiencing a temporal decrease in succinate concentration is equal to the proportion of those experiencing a temporal increase, (*p* = 3.83 × 10^−6^, two-tailed hypothesis test, assuming a binomial distribution with *n* = 161 trials with probability of 0.5 in each trial). This suggests that the chemotactic response depends in part on the absolute chemoattractant concentration experienced by cells^57–59^. In both cases, ‘correct’ repolarisation events (green bars) were significantly more abundant than ‘incorrect’ ones (magenta bars). Specifically, we rejected the null hypothesis that incorrect and correct repolarisation events occurred with equal frequency when the concentration was increasing (*p* = 1.98 × 10^−4^, one-tailed hypothesis test assuming a binomial process with *n* = 51 trials and probability of 0.5 in each trial) and for when the concentration was decreasing (*p* = 8.01 × 10^−20^, one-tailed hypothesis test assuming a binomial distribution with *n* = 110 trials and probability of 0.5 in each trial). Lastly, we note that cells experiencing a decrease in succinate concentration were significantly more likely to perform correct reversals than those experiencing an increase in succinate concentration. This analysis used a two-tailed Fisher′s exact test to reject the null hypothesis that there was no association between the sign of the temporal succinate gradient and whether the repolarisation event was correct or incorrect (*p* = 0.024). Source data provided as a Source Data file. Source data

Across three bio-replicates, we identified 171 cells that were stationary following the change in gradient orientation and subsequently underwent repolarization events (Fig. 4e, Supplementary Videos 1–16 and Supplementary Table 1; a detailed description of how repolarization events were identified is given in the Methods). A fraction of stationary cells sometimes began to move off after the gradient changed direction before observably altering their PilT–YFP distribution, so we also used cell movement to diagnose the chemotactic response of these initially stationary cells (Methods). Separating these 171 repolarization events by direction revealed a striking result: correct repolarization events occurred approximately 6 times more frequently than incorrect ones (148 correct, 23 incorrect; Fig. 4e), suggesting therefore that twitching cells directly sense chemoattractant gradients across the length of their cell bodies. This trend is remarkably consistent across stationary cells regardless of whether their initial PilT–YFP localization is nonpolar, unipolar or bipolar (Fig. 4e). Moreover, cells were observed to correctly determine the direction of the succinate gradient despite being subjected to sharp changes in succinate concentration over time (Fig. 5a–c, Extended Data Fig. 8, Supplementary Videos 1–16 and Supplementary Table 1). These temporal changes in concentration were two to three orders of magnitude larger than those in the Taylor–Aris dispersion experiments, indicating that spatial sensing is robust to large temporal changes in concentration, such as the random fluctuations that arise from twitching cell’s jerky movement relative to a chemical gradient. Last, we note that twitching *P. aeruginosa* cells always show a basal level of reversals even in the absence of chemical gradients^33^, which means that a proportion of incorrect repolarization events are expected, albeit at a lower frequency than correct ones (Figs. 4d,e and 5d).

**Extended Data Fig. 9:**
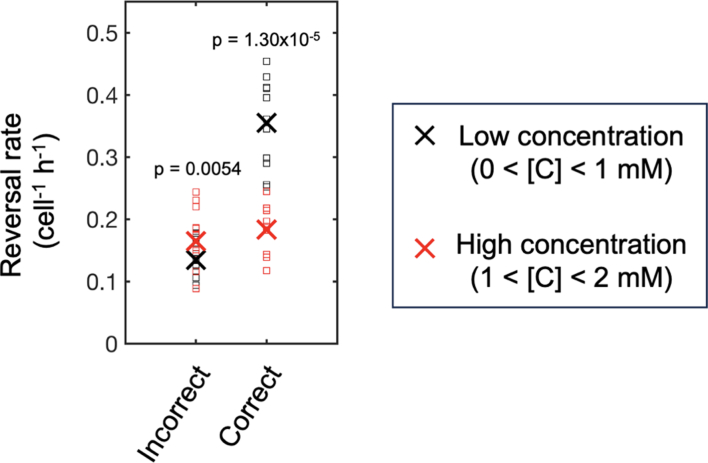
The chemotactic response of twitching cells is strongest at lower absolute concentrations. Theoretical predictions suggest that the chemotactic response magnitude will increase with gradient strength (which we have previously observed for twitching cells^33^,) and decrease with absolute concentration (see main text, and see^57–59^). Using datasets from twitching cells in our standard dual-flow chemotaxis assays (see Extended Data Fig. 1), we binned up cell trajectories according to whether they were in the low (0 < *C* < 1 m, black markers; squares represent individual bio-replicates) or high (1 < *C* < 2 mM, red markers) concentration region of the gradient. The ‘x’ symbols show the mean of each across all bio-replicates. Note that because the gradient is symmetrical about *C* = 1 mM, the cells in these two regions experience equivalent gradient strengths. Despite being exposed to equivalent gradient strengths, the cells in the higher concentration region have a significantly higher rate of incorrect reversals and a significantly lower rate of correct reversals than cells in the lower concentration region, (*p*-values show the results of two-sided paired *t*-tests). As in Extended Data Fig. 1b, this analysis only includes trajectory time-points that occurred before 5 h (that is, before the cell density becomes too high to reliably track cells in these experiments) and above a minimum gradient strength of 0.0006 mM µm^−1^ to ensure that cells within regions of the device where there is a very small spatial gradient were not included in our analysis. Source data provided as a Source Data file. Source data

The temporal changes did produce interesting trends, however. We observed more repolarization events in cells that experienced a sudden decrease in succinate concentration compared to those experiencing an increase in concentration (Extended Data Fig. 8). These findings are broadly consistent with previous work showing that the likelihood of responding to a stimulus increases when the background levels of that stimulus are lower (for example, a prediction of Weber’s law or receptor saturation kinetics^57–59^). In our alternating gradient experiments, we observed that more cells responded to the new gradient direction when they were experiencing a lower absolute concentration of succinate. We observed a similar pattern in our standard chemotaxis assays; that is, for a given gradient strength, cells are more likely to undergo correct reversals (and less likely to undergo incorrect reversals) when the absolute concentration of succinate was lower (Extended Data Fig. 9). However, while background concentration influences the response, we still found that correct repolarization events outnumbered incorrect repolarization events regardless of the background concentration that cells were exposed to. Specifically, in the alternating gradient experiments, correct reversals outnumbered incorrect ones by approximately tenfold when the concentration was decreasing, whereas an approximately fourfold difference was observed when the concentration was increasing (Extended Data Fig. 8). These results suggest that cells can correctly identify the direction of the spatial gradient across the lengths of their bodies across a range of absolute concentrations and regardless of the sign of the temporal gradient. Taken together, our data suggest that *P. aeruginosa* cells can robustly navigate chemoattractant gradients using spatial sensing.

## Discussion

**Extended Data Fig. 10:**
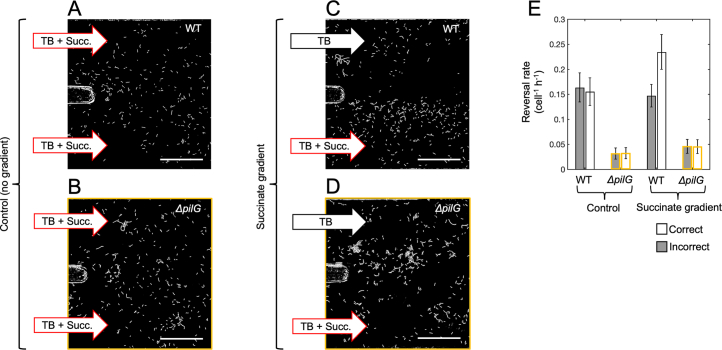
Cells lacking the response regulator, PilG, retain some degree of motility in microfluidic experiments^33^, but reverse at a lower rate than WT cells and can no longer bias reversals towards succinate. (**a**, **b**, **c**, **d**) Surface-attached *P. aeruginosa* cells (shown in white) within dual-inlet microfluidic devices (scale bars = 100 µm) were exposed to controls with a constant succinate concentration (*C* = 2 mM, A,B) or to succinate gradients (*C*_MAX_ = 2 mM, C,D) for *t* = 10 h. In gradients, WT cells undergo chemotaxis and accumulate at high succinate concentrations, whilst this accumulation is absent in no gradient controls. *P. aeruginosa* cells lacking *pilG* (images with yellow outline) cannot respond to succinate gradients and do not accumulate in either the succinate gradient or in the control. (**e**) In gradient free controls, *ΔpilG* cells (yellow outline) reverse direction less frequently than WT cells (black outline), but both WT and *ΔpilG* cells were equally likely to reverse direction no matter if they were initially moving in a downwards (‘incorrect’ reversals, grey bars) or upwards (‘correct’ reversals, white bars) direction. A one-sided exact Poisson test (**Methods**) yielded *p* = 0.93 and *p* = 0.87 across a total of *n* = 129 and 38 reversals for WT and *ΔpilG* cells respectively. However, in the presence of a succinate gradient, WT reversal rates were significantly larger when cells were initially moving towards decreasing succinate concentrations (‘correct’ reversals, white bars; *p* = 0.00072, *n* = 218, see also Extended Data Fig. 1). In contrast, the reversal rates of *ΔpilG* cells that were initially moving up or down the succinate gradient were not significantly different (*p* ≈ 1, *n* = 50). Error bars show 95% confidence intervals centred on the mean reversal rates assuming that reversals follow a Poisson distribution (**Methods**). Data is representative of three biological repeats. As in Extended Data Fig. 1b, this analysis includes trajectories from cells that were exposed to a succinate gradient >0.0006 mM µm^−1^ to exclude cells exposed to only very small spatial succinate gradients. Here tryptone broth is ‘TB’ and succinate is ‘Succ’. Source data provided as a Source Data file. Source data

We find that surface-attached *P. aeruginosa* cells can directly measure differences in concentration over the length of their bodies. By contrast, the signal transduction systems that guide chemotaxis in diverse swimming bacteria, including *P. aeruginosa*, use temporal sensing^20,24,29^. The use of spatial sensing was previously thought to be confined to the sophisticated signal transduction systems of eukaryotic cells^8,25^. Eukaryotic spatial sensing is regulated by a molecular ‘compass’ composed of intracellular chemical gradients. These gradients are generated from competition between rapid excitatory signalling generated by chemoeffector–chemoreceptor binding and slower, cell-wide inhibitory signalling, known as localized excitation, global inhibition or LEGI interactions^60,61^. It has recently been demonstrated that twitching *P. aeruginosa* cells are able to sense differences in mechanical stimuli across the lengths of their bodies via the two response regulators of the Pil-Chp chemotaxis-like system (PilG and PilH, which may prove comparable to the eukaryotic-like LEGI system^41^). We find here that PilG is also required for twitching chemotaxis towards succinate (Extended Data Fig. 10), and it is therefore possible that similar LEGI interactions could facilitate spatial measurements of chemical gradients in *P. aeruginosa*. We also note that the putative chemoreceptor of the Pil-Chp chemosensory system (PilJ) localizes to both cell poles in *P. aeruginosa*^62^, which could potentially facilitate spatial measurements.

Bacteria commonly live on surfaces, where they often experience strong and stable chemical gradients generated by a combination of molecular diffusion, nutrient consumption and the secretion of compounds from both nearby groups of bacteria and other organisms^63–68^. For example, it has recently been demonstrated that *P. aeruginosa* cells use pili to navigate towards compounds produced by nearby *Staphylococcus aureus* microcolonies and subsequently inhibit *S. aureus* growth^69^. Our results show that the well-established paradigm of bacterial chemotaxis, based on measuring changes in concentration over time, does not hold for surface-based movement in *P. aeruginosa*. Instead, we find that cells navigate on surfaces using spatial information. This mode of sensing is well suited to the slow movement and steep chemical gradients associated with living on surfaces and, relative to temporal sensing, it likely would allow twitching cells to measure larger changes in concentration, enhancing their ability to discriminate chemical gradients from stochastic noise (Fig. 1, Supplementary Information and refs. ^21,27,70^). Indeed, our experiments show that even stationary cells can use spatial information to sense chemical gradients. This observation raises the possibility that static bacteria living in mature biofilms could use the multiple, opposing chemical gradients that often form within biofilms^71^ to guide biofilm development.

## Methods

### Bacterial strains and culturing

Wild-type *P. aeruginosa* PAO1 (Kolter collection, ZK2019) was used as the model organism for this study. To visualize the localization of PilT within cells, we sought to express a fluorescently labelled copy of PilT from the native promoter of *pilT* on the chromosome. However, we were not able to detect any fusion protein using this approach with epi-fluorescent imaging, presumably because the native expression levels of *pilT* were too low. We therefore sought an alternative solution. First, we generated a *pilT* mutant lacking the first portion of the gene’s coding region in our model PAO1 strain using a previously published plasmid kindly gifted to us for this study (pJB203^55^; we refer to this mutant as *ΔpilT*). We then generated a PilT–YFP protein fusion expressed from a low-expression promoter (BG35) previously characterized in *Pseudomonas putida*^72^. Briefly, *pilT* was amplified from the chromosome of PAO1 using two primers that were complementary to the sequence immediately downstream of the *pilT* start codon (PILT_F) and ~100 base pairs downstream of the *pilT* stop codon (PILT_R; see Table 1 for primer sequences). The coding sequence of YFP was amplified from the plasmid pEYFP-N1 (Clontech) using an upstream primer (YFP_F) that additionally introduced the BG35 promoter immediately upstream of a ribosome binding site (designed using automated methodology described by ref. ^73^) and a downstream primer (YFP_R) that introduced a rigid linker^74^ to separate the functional domains of the two amplified proteins (YFP and PilT). These two amplified fragments were then combined by secondary PCR, ligated into the linearized vector pGEM-T (Promega) and transformed via electroporation into *E. coli* S17-1, a broad-host-range donor strain. We then used a previously established protocol for using a mini-Tn7 system to insert our *pilT-yfp* construct into the chromosome of our *ΔpilT* strain at its single *att*Tn7 site (ref. ^75^; *ΔpilT att*Tn7::*pilT-yfp*). Doing so restored the motility of our *ΔpilT* strain to wild-type levels, thus confirming that our PilT–YFP fusion protein is functional when expressed from the BG35 promoter at the chromosomal *att*Tn7 site (Extended Data Fig. 7). The final construct was confirmed by sequencing.

**Table 1:** Sequences of primers used in this study

Primer	Sequence
PILT_F	GCGGCAGCTAAGGCTGATATTACCGAGCTGCTCGCCT
PILT_R	CGCCGGCGTGATGTTCTCGCTCACTCAGGG
YFP_F	GCGGCCGCTTTATTTGACATGCGTGATGTTTAGAATTATAATTTGGGGA
	AGCCATCGGTACTATAAGGAGGTAAGTATGGTGAGCAAGGGCGAGGA
YFP_R	AGCCTTAGCTGCCGCCTCCTTAGCCGCAGCTTCAGCCAGCTCGTCCATGCCGAGA

All strains were grown from frozen stocks overnight in Luria–Bertani (Lennox) broth (Fisher, 37 °C, 250 r.p.m.) and sub-cultured (1:30 dilution) in tryptone broth (TB, 10 g l^−1^, Bacto tryptone) for 2.5 h to obtain cells in exponential phase. Cells were then diluted to an optical density at 600 nm of either 0.15 (experiment shown in Fig. 2 and Extended Data Figs. 2–5) or 0.5 (all other experiments) in TB media before being used to inoculate microfluidic experiments.

### Imaging

In the Taylor–Aris dispersion experiments (Fig. 2 and Extended Data Figs. 2–6), we used a Nikon Ti2-E inverted microscope equipped with a ‘Perfect Focus’ system and a Hamamatsu Orca-Fusion camera. For the experiment shown in Extended Data Figs. 1, 7 and 10, we used a Nikon Ti-E inverted microscope equipped with a ‘Perfect Focus’ system, a Hamamatsu Flash 4.0 v2 camera and a CoolLED pE-4000 illuminator. For the experiments that quantified the distribution of PilT–YFP (Figs. 3a–c, 4 and 5 and Extended Data Fig. 8), we used a Zeiss Axio Observer inverted microscope equipped with a ‘Definite Focus’ system, a Zeiss AxioCam MRm camera, and a Zeiss HXP 120 illuminator. We used ×20 Plan Apochromat air objectives throughout, except for our studies of the subcellular localization of our PilT–YFP fusion protein, which used a ×63 Plan Apochromat oil-immersion objective (on the Zeiss system). Time lapse images were collected using the Zen Blue 2012 (Zeiss) and NIS-Elements AR v4.51.01 (Nikon) software on the Zeiss and Nikon systems, respectively.

### Microfluidic experiments

Our custom-designed devices were cast with polydimethylsiloxane (PDMS) (Sylgard 184, Dow Corning) using moulds fabricated from SU-8 on silicon wafers (FlowJEM). Holes for tubing were punched through the PDMS using a Harris Unicore 1.5 mm biopsy tool (Agar Scientific). The PDMS was then bonded to glass coverslips (50 mm by 75 mm, number 1.5 thickness, Agar Scientific) using a corona treater (BD-20AC, Electro-Technic Products), as previously described^76^.

We plumbed the inlets and outlets of our microfluidic devices using Tygon microbore tubing (1.5 mm outside diameter) and then placed the entire set-up in a vacuum chamber for 1 h to reduce the potential for air bubbles. The devices were then mounted onto the microscope, and the outlet tubing was connected to a 10 ml plastic syringe (Luer-Lok, Becton Dickinson) using a 23-gauge needle (PrecisionGlide, Becton Dickinson). The syringe was filled with nutrient media (TB) and mounted onto a syringe pump (PhD Ultra, Harvard Apparatus). To remove air from the system, we first injected TB through the device at a flow rate of 100 µl min^−1^. Exponential-phase cells (as described above) were then drawn into the device via suction at a flow rate of 50 µl min^−1^ through the inlet tubing. Once cells reached the test section of the channel, all inlets and outlets were clamped using haemostats for 10 min, which allowed cells to attach in the absence of any flow. After this attachment period, the TB from the syringe was injected through the device at 100 µl min^−1^ for 10 min to remove any remaining planktonic cells. Last, the ends of the inlet tubing were placed into new reservoirs, and fluid was pulled through the device via suction for the remainder of the experiments.

The experiments shown in Extended Data Figs. 1, 7 and 10 were performed using the commercial BioFlux 200 microfluidic system (Fluxion Biosciences), using protocols that have been previously described^33^. We used our previously described model to quantify the chemical gradients within this device^77^.

### Taylor–Aris dispersion microfluidic experiments

For the experiments shown in Fig. 2 and Extended Data Figs. 2–5, we used a custom microfluidic device with a single inlet and outlet at either end of a rectangular microfluidic channel (30 mm in length with a cross section 1 mm wide and 75 µm deep). The inlet was connected to a 2 m length of Tygon tubing whose other end was placed in a reservoir containing TB mixed with succinate, and the entire system was filled with this fluid. Subsequently, we moved the end of the tube to another reservoir, containing a different concentration of succinate. When this new fluid was drawn into the tube via suction, Taylor–Aris dispersion mixed the interface between the media containing the two different concentrations of succinate longitudinally along the length of the 2 m tube before it flowed over the top of the cells. Alternatively, for control experiments, the end of the inlet tube was inserted into reservoirs that both contained TB with 1 mM succinate. Thus, cells in these control experiments did not experience any chemical gradients.

As discussed in the main text, our Taylor–Aris dispersion experiments were designed to expose cells to approximately the same mean concentration (*C*) and temporal concentration gradient (d*C*/d*t*) that cells experienced in the dual-flow experiments where pili-based chemotaxis towards succinate was readily observed (Extended Data Fig. 1 and ref. ^33^). In these experiments, the static spatial gradient of succinate had a magnitude of approximately d*C*/d*x* = 0.02 mM μm^−1^. Individual twitching cells moved along this gradient with an average speed of *V*_C_ = 0.2 μm min^−1^ (Extended Data Fig. 1c) and thus experienced a temporal gradient of succinate on the order of d*C*/d*t* = *V*_C_ d*C*/d*x* = (0.2 μm min^−1^) × (0.02 mM μm^−1^) = 0.004 mM min^−1^. Cells in this region of the device experienced an absolute concentration of succinate of *C* ≈ 1 mM.

Compared to flagella-based swimming, the motility of surface-attached *P. aeruginosa* cells is relatively slow, and reversals are relatively rare—on average, a cell reverses direction only once every several hours^33^. To ensure that our results were statistically robust, we aimed to collect as many cell trajectories (and thus reversals) as possible over the course of a Taylor-Aris dispersion experiment. To achieve this, we first used an automated microscope stage to simultaneously image 6 different fields of view within each microfluidic channel every minute (a total of 12 different scenes as we imaged in two channels simultaneously). Second, we aimed to expose cells to a temporal change in succinate concentration that lasted a period of approximately 1 h, so that we could detect a sufficient number of reversals over this period (labelled *t*_2_ in Fig. 2b–d and Extended Data Figs. 4 and 5).

The length scale of the succinate gradient that forms along the length of the inlet tube is set by competition between molecular diffusion in the radial direction and differential advection in the longitudinal direction of the tube, such that the length scale of the gradient in the tube increases with the flow rate. To obtain succinate gradients with the correct magnitude, we used previously described theory^44^ to design our experimental procedure. We first inserted the end of the inlet tube into the reservoir containing succinate at the higher concentration, *C*_MAX_, and then filled the entire microfluidic system with this media via suction. Then we switched the inlet tube to the reservoir containing the lower succinate concentration, *C*_MIN_, and pulled this second media into the inlet tube at a rate of 20 μl min^−1^ for 10 min. This formed a succinate gradient within the tube leading to the microfluidic device. We then lowered the flow rate on our syringe pump to 2 μl min^−1^ for the remainder of the experiment. The recently attached cells were allowed to adapt to the surface for approximately 2 h (under a continuous flow rate of 2 μl min^−1^) before the baseline measurements were recorded (white region labelled *t*_1_ in Fig. 2b,c).

We observed that the succinate gradient took approximately *τ* = 60 min to pass through the microfluidic channel, as visualized by using dye (Chicago Sky Blue 6B, 0.5 mg ml^−1^) in each run of the experiment (for example, Fig. 2b). This dye does not affect pili-based chemotaxis in *P. aeruginosa*^33^ and is predicted to have approximately the same distribution as the succinate given that they both have a similar molecular weight. We chose *C*_MAX_ = 1.16 mM and *C*_MIN_ = 0.84 mM, which yielded a d*C*/d*t* ≈ (*C*_MAX_ − *C*_MIN_)/*τ* = (1.16 mM − 0.84 mM)/60 min = 0.005 mM min^−1^ and ensured that cells experienced an average concentration of 1 mM succinate over the course of the experiment, which also matched the uniform succinate concentration used in control experiments. Our Taylor–Aris dispersion experiments thus closely matched the mean temporal gradient and mean concentration of succinate observed in the previously described dual-flow experiments (d*C*/d*t* ≈ 0.004 mM min^−1^ and *C* ≈ 1 mM, respectively).

The cells in our Taylor–Aris dispersion experiment primarily experience temporal variations in concentration that result from the spatial gradient of succinate flowing past them. We note that the speed of cells in our experiment *V*_C_ = 0.2 μm min^−1^ (Extended Data Fig. 1c) is orders of magnitude smaller than the speed at which the succinate gradient passes through the device (approximately 27,000 μm min^−1^), so a cell’s movement relative to the gradient has no appreciable impact on the temporal variation in succinate concentration they experience. Moreover, the length scale of the succinate gradient when it passes through the test section of the microfluidic device is approximately *L* = (27,000 μm min^−1^) × (60 min) = 1.6 m. Thus, the spatial gradient of succinate that cells experience across the length of their bodies in the Taylor–Aris dispersion experiments can be estimated as d*C*/d*x* ≈ (*C*_MAX_ − *C*_MIN_)/*L* = (1.16 mM − 0.84 mM)/1.6 m = 2.0 × 10^−7^ mM µm^−1^, which is several orders of magnitude smaller than the spatial gradients that cells experienced in the dual-flow experiments (d*C*/d*x* ≈ 0.02 mM μm^−1^).

In summary, the cells in the Taylor–Aris dispersion experiments experience approximately the same mean temporal stimuli as they do in the previous dual-flow experiments, while experiencing spatial gradients that are only vanishingly small in comparison.

To follow cell motility in these experiments, images were captured in brightfield at a rate of 1 frame per min. Using Fiji (v2.0.0)^78^, we stabilized the time series of brightfield images using the Image Stabiliser plugin to remove drift in the *x*, *y* plane. Next, the background was made more homogenous using the Normalise Local Contrast plugin, and the intensity of the background was reduced using the Subtract Background feature. Finally, a bleach correction plugin was used to correct for long-term changes in the relative pixel intensity of the cells in brightfield compared to the background, which varies as the concentration of dye changes over time^79^. Cells were then tracked using the Trackmate (v2.3.0) plugin for Fiji (v1.5.4)^80^. Finally, to analyse cell motility and to detect when cells reverse direction, we used an image analysis pipeline in Matlab (2019b) that we developed previously to study twitching motility in *P. aeruginosa*^33^.

### Cell responses to sharp temporal changes in concentration

Twitching motility is characteristically jerky, and cells frequently undergo rapid displacements caused by the release of single pili, causing them to briefly move ~20 times faster than their average speed^38,50^. While these rapid displacements constitute a relatively small fraction of a cell’s total movement time, their contribution to a cell’s total displacement is approximately equal to their slower and steadier form of movement^50^. As noted in the main text, the temporal gradient that a cell experiences is linearly proportional to its movement speed (as d*C*/d*t* = *V*_*C*_ d*C*/d*x*), and so a cell is predicted to experience temporal gradients that are ~20 times larger during these rapid displacement events. We thus tested the possibility that twitching cells in the presence of chemical gradients might employ a temporal sensing modality that is tuned to respond to these relatively short but steep temporal chemoattractant gradients.

For these experiments, we used a dual-inlet BioFlux 200 microfluidic system (Fluxion Biosciences) in which one inlet was connected to TB mixed with a larger concentration of succinate (*C*_MAX_ = 1.16 mM), while the other inlet was connected to TB mixed with a smaller concentration of succinate (*C*_MIN_ = 0.84 mM). Instead of passing fluid through both inlets simultaneously so they formed a spatial gradient within the test section^33^, we instead passed fluid through only one inlet at a time, which exposes all cells in the test section to the same succinate concentration. We used computer-controlled software to alternate the flow between the two inlets, such that cells sequentially experienced a rapid increase in succinate concentration followed by a rapid decrease in succinate concentration over time. Like the Taylor–Aris dispersion experiments described in the previous section, we chose these *C*_MAX_ and *C*_MIN_ values so that the mean succinate concentration that cells experienced was 1 mM, which was the concentration where chemotaxis was observed to peak in the dual-flow experiment where cells where exposed to a spatial gradient of succinate.

We added Chicago Sky Blue 6B dye (0.5 mg ml^−1^) to the media containing the higher concentration of succinate (*C*_MAX_), whereas the media containing the lower concentration of succinate (*C*_MIN_) did not contain dye. By quantifying the change in dye intensity at the downstream end of the test section of the device, we observed that cells experienced a smooth change in concentration between the two different media over a timescale of *τ* ≈ 1.5 min (Extended Data Fig. 6). Because the time period of the temporal gradient (*τ*) in these experiments is relatively short and therefore affords less time to observe reversals, we alternated the flow between the two inlets every 15 min so that we could expose cells to at least six increases and decreases in succinate concentration over the course of one experiment (Extended Data Fig. 6a). We observe that the transition between the two succinate concentrations occurs smoothly and consistently in the test section of the device. We note that the overall duration of our microfluidic experiments is limited because in situ cell division eventually crowds the surface, which makes tracking individual cells difficult.

We can estimate the temporal gradient in these experiments as d*C*/d*t* ≈ (*C*_MAX_ − *C*_MIN_)/*τ* = (1.16 mM − 0.84 mM)/1.5 min = 0.2 mM min^−1^; Extended Data Fig. 6b,c), which is one to two orders of magnitude larger than the temporal gradients that cells were exposed to in the Taylor–Aris dispersion experiments described in the previous section and is approximately the same strength as the temporal stimuli that we predict a cell in our dual-flow experiments will experience momentarily during pili release events^38,50^. We can estimate the spatial gradients that form over the length of the test section in these experiments as d*C*/d*x* ≈ (*C*_MAX_ − *C*_MIN_)/(*Uτ*) = (1.16 mM − 0.84 mM)/(2,500 µm min^−1^ × 1.5 min) = 8.5 × 10^−5^ mM µm^−1^, (where *U* is the mean flow speed), which is approximately 200-fold smaller than the spatial gradients that cells experienced in the dual-flow experiments (d*C*/d*x* ≈ 0.02 mM μm^−1^).

To follow cell motility, two fields of view were imaged in brightfield at a higher frame rate of 7.5 frames per min. Using Fiji^78^, images were processed and tracked using the Trackmate plugin^80^ as described above. To analyse cell motility and to detect when cells reverse direction, we once again used our previously developed image analysis pipeline in Matlab^33^.

To test whether cells can sense and respond to this larger temporal stimulus, we compared cell reversal rates before, during and after they experienced a temporal gradient in succinate concentration across six increases and six decreases in succinate concentration (Extended Data Fig. 6b,d). Our statistical analyses found that neither an increase nor a decrease in succinate concentration elicited cells to change their reversal rate (Extended Data Fig. 6c,e). These experiments thus show that surface-attached *P. aeruginosa* cells do not respond to the larger temporal gradients that they would experience during pili release events.

### Quantifying PilT–YFP sub-cellular localization

To measure how the localization of our PilT–YFP fusion protein varies from a cell’s leading pole to its lagging pole, we developed an image analysis pipeline that automatically tracks cell position, length and orientation in brightfield and uses this information to quantify the distribution of YFP using the corresponding epi-fluorescence images. Brightfield images were captured at a frame rate of 7.5 frames per min, while epi-fluorescence images to visualize YFP were simultaneously acquired at a lower frame rate of 0.5 frame per min. The higher frame rate for brightfield allowed us to track cell motility with sufficient accuracy, whereas the lower frame rate for the YFP imaging allowed us to avoid bleaching and phototoxicity.

All preliminary image analysis was conducted in Fiji^78^. Brightfield images were processed as outlined above. Epi-fluorescence images were processed in the same way as brightfield images, except we additionally used a Difference of Gaussian filter to enhance the contrast of the localized accumulations of PilT–YFP.

The cells in these processed images were then tracked using software called the Feature-Assisted Segmenter/Tracker (FAST v2.1^81^; https://mackdurham.group.shef.ac.uk/FAST_DokuWiki/dokuwiki/doku.php?id=start) which allowed us to track cell position and orientation with greater precision compared to the tracking plugins available in Fiji. To map how the distribution of PilT–YFP varies along the cell length and how that distribution changes as cells move, we used FAST to calculate the cell centroid, length and orientation of each cell in the brightfield images. We then used this information to calculate the position of the ‘centreline’ of each cell (that is, a line that passes through the middle of a cell along its major axis) on the corresponding YFP epi-fluorescence image. However, PilT–YFP localizations do not always occur exactly along the predicted centreline—but rather they were sometimes found slightly to one side of the centreline. Thus, to accurately quantify the distribution of the fusion protein, we needed to develop a method that could detect PilT–YFP localizations even when they were offset slightly from the cell’s centreline, in addition to being robust to small amounts of cell movement that occurred in the time interval between when the brightfield and YFP images were captured. To account for these factors, we used the Matlab function ‘improfile’ to calculate the YFP fluorescence intensity along a series of 10 parallel lines with the same orientation and length as a cell but separated by a small distance (0.09 µm) from one another so that collectively they spanned a width approximately equal to the width of the cells (~0.9 µm). We then calculated the maximum YFP intensity at fixed intervals along the length of these ten lines to obtain the maximum fluorescence intensity at each position along the cell’s length. This process was used to record the distribution of PilT–YFP along the length of each cell at every time point across the three bio-replicate experiments (*n* = 52,179 trajectory points).

A small number of cell fragments and other detritus were occasionally observed in the brightfield images we used to segment cells; however, these generally were not visible on the corresponding YFP images. To prevent these from inadvertently being included in our analyses, we measured the mean YFP intensity of all objects using the segmentations obtained from the brightfield image and removed trajectories without appreciable YFP signal from subsequent analyses. We also omitted any cells with an aspect ratio smaller than 1.4, which ensured that our analyses only included cells that were attached to the surface by both cell poles.

We next quantified the distribution of PilT–YFP fusion protein within the poles of the cells. Because the maximum YFP intensity often does not occur at the very tip of the pole, we measured the maximum YFP intensity in the vicinity of the poles. The cell length was measured using YFP images, and the maximum YFP intensity was calculated in the two regions at either end of the cell, each corresponding to one tenth of the overall cell length. To classify the distribution of PilT–YFP within a cell as nonpolar, unipolar or bipolar (Fig. 3), we normalized the maximum YFP intensity within each pole by the mean YFP intensity in the central one fourth of the cell. If the normalized YFP intensity in a given pole (denoted as *I*_1_ and *I*_2_ for pole 1 and pole 2) exceeded a threshold *I*_MIN_, the protein was considered to have aggregated within that pole. More specifically, if both *I*_1_ > *I*_MIN_ and *I*_2_ > *I*_MIN_, the cell was considered bipolar, whereas if either *I*_1_ > *I*_MIN_ or *I*_2_ > *I*_MIN_, the cell was considered unipolar. Last, if *I*_1_ < *I*_MIN_ and *I*_2_ < *I*_MIN_, the cell was considered nonpolar. To determine the value of *I*_MIN_ for a given bio-replicate, we calculated the normalized YFP intensity values (*I*_1_ and *I*_2_) for all cells in a YFP image, which had been processed as described above. This allowed us to choose an *I*_MIN_ value by visual inspection that correctly distinguished cell poles with PilT–YFP localization from those that lacked PilT–YFP localization.

To increase the accuracy of the automated assignment of cells as bipolar, unipolar or nonpolar, we also implemented the following two rules:

- When *P. aeruginosa* nears cell division, the pili machinery (and thus the PilT–YFP protein fusion) begins to localize additionally to the nascent cell poles, which are positioned at mid-cell^82^. In such instances, the maximum fluorescence intensity can occur in the mid-cell region rather than at the poles. As we are interested in the processes underlying cell motility (rather than cell division), we excluded cells from our analyses whose average fluorescence in the middle one fourth of their bodies was larger than that found in either of the poles.
- Some cells that were initially assigned as bipolar (that is, because the YFP intensity at its two poles, *I*_1_ and *I*_2_, both exceeded *I*_MIN_) were re-assigned as unipolar if the YFP signal in one of their poles was much stronger than in their opposite pole. To detect such instances, we plotted the ratio of *I*_1_ and *I*_2_, dividing the larger YFP intensity by the smaller one, for each cell in our processed YFP images. We then used the threshold that best distinguished bipolar cells from unipolar ones by direct visual inspection. This allowed us to ensure that we assigned cells with strongly asymmetrical patterns of PilT–YFP localization as ‘unipolar’, rather than ‘bipolar’.

To compare the distribution of PilT–YFP in stationary and moving cells, we classified trajectories by their speed (Fig. 3). Due to pixel noise and the effect of fluid flow, the measured trajectories of non-motile cells showed a finite velocity. To account for these effects, we classified cells moving slower than 0.038 μm min^−1^ as ‘stationary’, whereas cells moving faster than this threshold were classified as ‘motile’. To prevent cells simply jostling back and forth from being considered motile, we additionally removed trajectories from the motile category whose net to gross displacement ratio (NGDR) was less than 0.04. In addition, we excluded cells that were actively rotating from the motile category by identifying cells whose bodies had an angular velocity larger than 0.073 radians min^−1^ for a contiguous period of longer than 2 min. These angular velocities were obtained from measurements of cell orientation that had been smoothed with a first-order Savitzky–Golay filter (using a 20 min window) to reduce noise. All the parameters used in these analyses were extensively ground-truthed to ensure that they had the desired effect.

### Generating alternating spatial chemoattractant gradients

To expose cells to a spatial chemoattractant gradient that alternates in direction by 180°, we used a custom microfluidic device described in detail previously^33^. Briefly, the device is composed of a Y-shaped channel with four inlets (two inlets in each branch of the Y) and a single outlet that was connected to a syringe pump.

In these experiments, a steady spatial gradient of succinate forms along the centreline of the device, where the fluids from two inlets located in opposite arms of the Y-shaped channel meet one another. The fluid supplied through one arm contained nutrient media (TB) supplemented with 2 mM of succinate and Chicago Sky Blue 6B dye (0.5 mg ml^−1^), whereas fluid from the other arm contained only undyed nutrient media. Molecular diffusion generated a stable gradient of succinate across the width of the channel, which could be readily quantified by imaging the dye as they have a similar diffusion coefficient.

The syringe pump pulled media through the device via suction (5 μl min^−1^) from reservoirs connected to the four inlets of the device. A haemostat was used to clamp the tubing connected to two of the inlets at any given time. To change the direction of the gradient, the haemostat is removed from one pair of tubes and transferred to the other pair, which contain the same two fluids but in the opposite orientation (see ref. ^33^ for details). We changed the direction of the gradient approximately every 45–60 min, and we monitored cells for repolarization events for as long as possible after the gradient swap, before we needed to pause the imaging to set up the next gradient swap.

Brightfield images were captured at a frame rate of 7.5 frames per min so that changes in the gradient and cell movement could be tracked at a high temporal resolution. Epifluorescence images of the cells were captured at a slower frame rate of 0.4 frame per min to avoid bleaching of the PilT–YFP fusion protein and to prevent phototoxicity. The details of how cells were tracked and how the distribution of the fusion protein inside them was quantified is outlined below.

### Analysing PilT–YFP localization in stationary cells

To directly test whether surface-attached *P. aeruginosa* cells are capable of spatial sensing, we exposed our *ΔpilT*::*pilT-yfp* strain to a spatial gradient of succinate that alternated direction using the microfluidic device outlined in the previous section. To exclude the possibility that cells could use temporal sensing to determine the orientation of the new succinate gradient, we only considered repolarization events that occurred in stationary cells (see main text). Because the PilT–YFP protein fusion tends to localize to a cell’s leading pole (Fig. 3c), each repolarization event can be categorized according to whether the new leading pole of a stationary cell is oriented towards (‘correct’) or away from (‘incorrect’) increasing succinate concentrations following the change in gradient orientation (Fig. 4). In addition, we also classified repolarization events according to whether PilT–YFP was initially localized in both poles (bipolar), in only one pole (unipolar) or in neither pole (nonpolar) before the repolarization event occurred (Fig. 3g–i).

While our other analyses used automated cell tracking to quantify cell behaviour, we decided to detect and classify these repolarization events manually for two main reasons. First, a relatively small number of repolarization events are observed in these experiments, so we wanted to follow the behaviour of every single cell and rigorously ground-truth all putative repolarization events to confirm that they were not erroneous. Secondly, many stationary cells reside in densely packed groups, which help to stifle movement. However, densely packed cells are challenging to track using automated methods without occasional errors, and it is difficult to measure an individual cell’s PilT–YFP distribution without inadvertently having it contaminated by the YFP signal produced by neighbouring cells. (Note that in other experiments that were analysed using automated cell tracking, we developed filters to specifically exclude cells that were clustered together.)

We analysed the behaviour of every cell that was visible in the 16 different fields of view collected over the course of three separate microfluidic experiments (Supplementary Videos 1–16) and classified them with a detailed set of rules outlined in the following four sub-sections below. Out of >1,000 cells that were investigated, we identified 171 stationary cells that performed a repolarization event—as defined by these rules—following the gradient swap. To prevent potential errors, a preliminary list of repolarization events was independently assessed by two co-authors (J.H.R.W. and W.M.D.), and any discrepancies were reconciled before our final analyses. All 171 repolarization events are labelled in Supplementary Videos 1–16, along with the details of how each was classified (Supplementary Table 1).

Below we describe in detail the rules that were used to define and classify each putative repolarization event.

#### Identifying when a repolarization event occurs

We search for potential repolarization events in cells that are stationary after the succinate gradient changes direction. In many cases, stationary cells first localize PilT–YFP exclusively to their new leading pole before moving off; however, sometimes cell movement occurs first. A repolarization event therefore occurs as soon as a stationary cell either (A) develops a unipolar pattern of PilT–YFP localization that is different from that of its initial localization of PilT–YFP or (B) moves off in a direction different from that of its initial localization of PilT–YFP. In the first case, (A), a cell must re-localize PilT–YFP to a single pole in at least two of four consecutive frames (10 min), whereas in the second case, (B), a cell must move off in a consistent direction for at least two frames at a speed corresponding to at least one cell width per frame.

Following a repolarization event, we define a cell’s ‘new leading pole’ as the one that either contains the new unipolar PilT–YFP localization or leads its initial movement, whichever has occurred first. The orientation of a cell’s ‘new leading pole’ after the repolarization event is then used to determine whether it can be classified as a ‘correct’ or ‘incorrect’ repolarization event by comparing its orientation relative to that of the new succinate gradient (Fig. 4a–c and Supplementary Table 1).

Importantly, for a repolarization event to have occurred, a cell must not have previously had a unipolar PilT–YFP localization in the ‘new leading pole’ in either two or more of the four frames (10 min) that precede the appearance of the new succinate gradient, or within the frame that immediately precedes the appearance of the new succinate gradient. This requirement thus ensures that cells have actively changed their distribution of PilT–YFP following the change in gradient direction and also prevents short-lived, random fluctuations in the distribution of PilT–YFP from being erroneously classified as a repolarization event.

#### Defining which cells are considered ‘stationary’

These experiments aim to analyse the behaviour of stationary cells because motile cells could potentially use temporal sensing to determine the orientation of the new succinate gradient. However, cells can sometimes show small amounts of movement that are unrelated to their motility. For example, the flow in our experiments tends to push cells downstream while cells at the periphery of densely packed cell clusters can get pushed radially outwards by their neighbours as the cluster grows. As such movements are not under the active control of a cell, they could not encode information about the direction of a gradient via temporal changes in succinate concentration in the same way that active motility would. In addition, in our experiments, cells that are pushed a small distance by flow tend to move in the direction orthogonal to the gradient and thus do not experience appreciable changes in succinate concentration over time. We therefore monitor a cell’s movement in the direction along the gradient to ensure it is sufficiently small in the period preceding a repolarization event.

To determine whether a cell can be considered stationary, we monitor its movement from the frame after the last frame in which the initial succinate gradient was present until the frame in which the cell undergoes a repolarization event. However, as some repolarization events occur shortly after the gradient has changed direction, we also monitor cell movement for at least three frames (7.5 min) before any putative repolarization event. A cell is then considered ‘stationary’ within these time periods provided that its centroid neither (a) moves more than half a cell width in the same direction for two consecutive frames nor (b) moves more than one cell width at any point. All distances are measured along the direction of the chemical gradient, and a cell width is approximately 0.9 µm.

Note that many cells are stationary for a finite period, and so a cell that is currently stationary will likely have moved at some point in the past. Our analyses include cells that move while the initial succinate gradient is still present but subsequently stop moving before the gradient starts to change direction. This is because such previous movement could not inform a cell that the orientation of the succinate gradient will change later in the experiment.

#### Assigning a cell’s polarity before repolarization events

We categorize repolarization events according to the PilT–YFP localization that they previously showed (Figs. 3g–i and 4e). For a repolarization event to be assigned as either nonpolar, unipolar or bipolar, the cell must have had that polarity mode more frequently than any other in the four frames (10 min) preceding the appearance of the final gradient orientation. If two different polarity modes are each present for two frames apiece, then we assign the polarity mode that occurs in the frame immediately preceding the appearance of the final gradient orientation. We note that the ‘initial polarity’ of two cells could not be resolved in these experiments because one of their poles was initially in very close proximity to that of their neighbours, which prevented us from distinguishing the YFP signal that belonged to each cell. Thus, the initial polarity of these two cells was classified as ‘not assignable’ in Supplementary Videos 1–16 and Supplementary Table 1.

We also observed a small number (*n* = 13) of repolarization events in newly divided cells. If a cell that is stationary (as defined above) divides shortly after the change in gradient orientation, one or both of the resulting daughter cells could in theory undergo a repolarization event (as defined above). In these cases, the distribution of PilT–YFP is assigned as nonpolar, unipolar or bipolar (Figs. 3g–i and 4e) according to the most frequent localization pattern in the frames between the cell division event and the subsequent repolarization event. We did not consider PilT–YFP localizations at the midpoint of the mother cell before cell division in our analyses, because they are not necessarily related to motility and can be asymmetrically divided between the two daughter cells during septation^82^.

#### Assigning temporal changes in succinate concentration

As above, we used Chicago Sky Blue 6B dye to visualize the alternating succinate gradient. When the gradient changes orientation by 180°, cells initially situated in regions of low succinate concentration (*C* < *C*_MAX_/2, as determined by the dye intensity) experience a temporal increase in succinate concentration, whereas those initially in regions of high succinate concentration (*C* > *C*_MAX_/2) experience a temporal decrease in succinate concentration. By following changes in the dye intensity, we were able to group repolarization events according to whether they occurred in cells that had experienced an overall increase or decrease in succinate concentration (Extended Data Fig. 8). However, it was very difficult to distinguish the small temporal changes in succinate concentration (and thus dye intensity) experienced by cells situated close to the centreline of the spatial gradient. These cells (*n* = 10) were therefore excluded from the analyses that compared the response of cells experiencing a step-up in succinate concentration to a step-down in succinate concentration. The ‘temporal change in [succinate]’ of these cells is marked as ‘not assignable’ in Supplementary Videos 1–16 and Supplementary Table 1.

### Methods used to illustrate repolarization events

We used automated cell tracking software (Trackmate plugin, Fiji^78,80^) to follow cell movement across four exemplar repolarization events to quantify changes in cell speed and to map changes in succinate concentration at the location of each of the four cells (Fig. 5a–d). To ensure that we could obtain trajectories that spanned the entire length of experiment, the cells of interest were cropped out frame by frame using the ‘Brush Tool’ included with Fiji (v2.0.0). This left us with only a single cell visible in the entire time series of images, ensuring the automated tracking was not influenced by the presence of neighbouring cells. We used the resulting curated trajectories to calculate a cell’s position relative to the chemoattractant gradient (grey lines in Fig. 5a–d). The concentration of succinate that a cell experienced over time (black lines in Fig. 5a–d) was quantified using the Chicago Sky Blue 6B dye, which was mixed with the 2 mM succinate solution. The distribution of dye was imaged using brightfield microscopy, and separate experiments showed a linear dependence between the pixel intensity and dye concentration, allowing us to easily estimate the succinate concentration at the position of each cell within the device.

### Statistical analyses

To test whether cells use temporal chemoattractant gradients to guide pili-based motility, we developed statistical methods to determine whether cells alter their reversal rate in response to temporal gradients of succinate in comparison to control conditions where the concentrations of succinate were constant. Our Taylor–Aris dispersion experiments (Fig. 2 and Extended Data Figs. 3–5) are ~3 h long, and the total number of cells changes over this timescale due to cell detachment from and attachment to the surface, as well as continued cell division (Extended Data Fig. 2 and ref. ^46^). Furthermore, even in the absence of any chemical gradients, reversal rates change over time, likely driven by cells undergoing physiological adaptation following surface attachment (refs. ^47,48^) (Extended Data Fig. 3). To take these temporal trends into account, we divided our datasets into three time bins corresponding to before, during and after the cells experienced a temporal gradient of succinate (see *t*_1_, *t*_2_ and *t*_3_ in Fig. 2 and Extended Data Figs. 4 and 5).

Our Taylor–Aris dispersion experiments imaged six different fields of view simultaneously at a frame rate of 1 frame per min, yielding several thousand trajectories at each time point (Extended Data Fig. 2a–c). However, reversals are relatively rare—on average a cell reverses direction only once every several hours. Our datasets therefore consist of a very large number of time points at each of which a cell can either carry on moving in a relatively straight line or, with a low probability, reverse direction. We therefore assume that reversals are Poisson distributed, allowing us to calculate the confidence intervals of our reversal rate estimates. Using this assumption, we also used the exact Poisson test (using the ‘poisson.test’ function (v3.6.2) in R (v4.2.3)) to test for differences in reversal rates between control and experimental conditions (Fig. 2 and Extended Data Figs. 4 and 5).

A similar approach was used to generate confidence intervals for our estimates of reversal rates for cells moving either up or down spatial chemoattractant gradients (Extended Data Figs. 7 and 10). However, in these analyses, we calculated the mean reversal rate using data from the entire experiment (rather than subdividing it into different bins in time), because in these experiments, the gradient was present for the entire duration.

### Strain availability

The bacterial strains used in this study are available from the corresponding authors upon request.

### Reporting summary

Further information on research design is available in the Nature Portfolio Reporting Summary linked to this article.

## Supplementary information

Supplementary Information Supplementary Discussion and Legends for Supplementary Videos 1–16.

Reporting Summary

Supplementary Video 1 Supplementary Video 1.

Supplementary Video 2 Supplementary Video 2.

Supplementary Video 3 Supplementary Video 3.

Supplementary Video 4 Supplementary Video 4.

Supplementary Video 5 Supplementary Video 5.

Supplementary Video 6 Supplementary Video 6.

Supplementary Video 7 Supplementary Video 7.

Supplementary Video 8 Supplementary Video 8.

Supplementary Video 9 Supplementary Video 9.

Supplementary Video 10 Supplementary Video 10.

Supplementary Video 11 Supplementary Video 11.

Supplementary Video 12 Supplementary Video 12.

Supplementary Video 13 Supplementary Video 13.

Supplementary Video 14 Supplementary Video 14.

Supplementary Video 15 Supplementary Video 15.

Supplementary Video 16 Supplementary Video 16.

Supplementary Table 1 Supplementary table detailing and indexing all repolarization events.

## Source data

Source Data Fig. 2 Statistical source data.

Source Data Fig. 3 Statistical source data.

Source Data Fig. 4 Statistical source data.

Source Data Fig. 5 Statistical source data.

Source Data Extended Data Fig. 1 Statistical source data.

Source Data Extended Data Fig. 2 Statistical source data.

Source Data Extended Data Fig. 3 Statistical source data.

Source Data Extended Data Fig. 4 Statistical source data.

Source Data Extended Data Fig. 5 Statistical source data.

Source Data Extended Data Fig. 6 Statistical source data.

Source Data Extended Data Fig. 7 Statistical source data.

Source Data Extended Data Fig. 8 Statistical source data.

Source Data Extended Data Fig. 9 Statistical source data.

Source Data Extended Data Fig. 10 Statistical source data.

## Data Availability

Source data are provided with this paper. Image data (~650 GB) is available from the corresponding authors upon request. All other data that support the findings of this study can be accessed at 10.15131/shef.data.25800409.

## Extended data

–

## Code availability

The code used to generate the findings of this study can be accessed at 10.15131/shef.data.25800409.
