# Supplementary material for: Individual bacterial cells can use spatial sensing of chemical gradients to direct chemotaxis on surfaces
Source: Nat Microbiol. 2024 Sep 2;9(9):2308–22. doi: 10.1038/s41564-024-01729-3 (PMC11371657; doi:10.1038/s41564-024-01729-3)
Supplement: Supplementary file 1 — Supplementary Discussion and Legends for Supplementary Videos 1–16. [file 41564_2024_1729_MOESM1_ESM.pdf]

nature microbiology

Supplementary information

https://doi.org/10.1038/s41564-024-01729-3

Individual bacterial cells can use spatial sensing of chemical gradients to direct chemotaxis on surfaces

In the format provided by the authors and unedited

Legend for movies 1-16

Each of the repolarisation events included in our analyses (n = 171) can be observed in the 16 different supplementary movies included in the appendix. In movies 1-9, we used dye (shown in false-colour blue) to label media containing no succinate (C_{MIN} = 0 mM), whilst in movies 10-16, we used dye to label media containing succinate (C_{MAX} = 2 mM). To guide the eye, the arrow shown at the bottom indicates the direction in which the succinate concentration increases in all movies. The flow moves vertically from top to bottom. The movie pauses on the frame before the succinate gradient changes direction to mark the cells that subsequently perform a repolarisation event. The shape of the symbol used to mark the cells corresponds to the cell's initial PilT-YFP polarity prior to the change in gradient orientation (see legend) and symbol colour corresponds to whether the repolarisation event is "correct" (green) or "incorrect" (magenta). A summary of each repolarisation event and how they were classified is provided in Supplementary Table 1 and the detailed set of rules that was used to detect and classify repolarisation events are outlined in the Methods. For clarity, we only show the YFP fluorescent images taken at 2.5 min intervals and have omitted the brightfield images that were taken at 8 sec intervals.

Supplementary discussion

Planktonic and surface-attached bacteria face fundamentally different constraints when sensing chemical gradients

Several arguments have been proposed to explain why swimming bacteria have evolved to use temporal sensing – rather than spatial sensing – to detect chemical gradients. However, those same arguments do not hold for surface-attached, twitching bacteria and in some cases, lead to the opposite conclusions:

1. Swimming cells can travel in a relatively straight line for only a few seconds before being reoriented by Brownian rotational diffusion [1, 2]. However, the rapid movement speed of swimming bacteria allows them to cover tens of body lengths over the timescale of a "run" allowing them to measure changes in concentration that occur over length scales much larger than the size of their bodies (**Fig. 1**). In contrast, surface-attached bacteria are not reoriented by Brownian rotational diffusion and so could in theory measure changes in concentration that occur over much longer timescales. Compared to swimming cells that swim at $V_C \approx 2000$ µm min^{-1} [3], twitching cells move four orders of magnitude more slowly ($V_C \approx 0.2$ µm min^{-1}) and typically traverse a distance equivalent to the length of their bodies (≈ 5 µm) in approximately 25 min. Thus, unless twitching bacteria have a very long memory, allowing them to measure changes in their chemical environment over periods >25 min (i.e. longer than their doubling time in optimal conditions [4]), spatial sensing would allow twitching cells to measure larger changes in concentration compared to temporal sensing. In contrast, the chemosensory systems of swimming bacteria are

typically tuned to respond to temporal changes in concentration that occur over timescales on the order of a few seconds, which is approximately the timescale over which they can swim in a straight line before being reoriented by Brownian rotational diffusion [5].

2. Berg and Purcell, 1977 [2] identified another obstacle to spatial sensing in rapidly swimming cells - as a cell moves through a chemoattractant field of uniform concentration, it will come into contact with more chemoattractant molecules at its front, compared to its rear. This creates an apparent "spatial gradient" across the cell length that would be challenging to disentangle from any external gradients. If a cell is assumed to be a perfectly absorbing sphere of radius, *a,* moving at constant velocity, V_C, Berg and Purcell find that the difference in the number of molecules that reach a cell's front and back hemisphere differs by a factor of approximately $1 + 3a\ V_C\ / D$, where D is the diffusion coefficient of a given chemoattractant. For a swimming cell, with an equivalent radius of a = 3 µm that moves at V_C = 2000 µm min^{-1} and responds to a small chemoattractant molecule in water (D ~10^{-9} m^2 s^{-1} = 60,000 $µm^2$ min^{-1}), it would experience a 1.3-fold larger flux of chemoattractant molecules to its front compared to its rear in the absence of any external chemical gradients. Whilst this apparent gradient would be reduced if the cell does not act as a perfect absorber, it is predicted to still pose a challenge if swimming cells were to use spatial sensing [2]. In contrast, twitching cells move four orders of magnitude more slowly and thus would only experience a 1.00003-fold difference. While there are hypothetical arguments for how swimming cells might be able to overcome these apparent spatial gradients [6], the much slower speed of twitching cells suggests that these apparent gradients would not pose a challenge for spatial sensing in surface-attached bacteria.

3. Several authors claim that bacterial cells are too small for spatial sensing [7-10]. Diffusion is predicted to smooth out any intracellular gradients in intracellular signalling proteins within seconds across the length of a micron-sized bacterial cell [11, 12]. Despite this limitation, bacteria have been shown to establish cytoplasmic gradients of protein phosphorylation by localising the kinase and phosphatase proteins that drive phosphorylation and de-phosphorylation to opposite cell poles [8]. Importantly, both the pili machinery (e.g., **Fig. 3** and [13, 14]) and the associated chemoreceptor (PilJ) of the Pil-Chp chemosensory system that regulates twitching motility [15] localise to the two cell poles, which could facilitate signal detection and processing across the length of a twitching cell.

4. Individual twitching cells tend to jerk back and forth as they move, owing to the stochastic detachment of individual pili [16, 17]. If twitching cells used temporal gradients to guide chemotaxis, they would therefore have to distinguish rapid temporal fluctuations (that

frequently change signs) from those that occur over longer timescales to ascertain whether they were moving up or down a chemical gradient. However, the chemosensory system used by swimming *E. coli* cells has been found to function as a bandpass filter that exhibits a maximal response for temporal stimuli with a timescale of approximately four seconds and a sharp cut-off for higher frequency stimuli [18]. This precedent suggests that the jerky motion of twitching bacteria does not in itself preclude temporal sensing, since the high frequency variations caused by jerky motion [17] could in principle be filtered by a chemosensory system that was tuned to preferentially respond to lower frequency temporal stimuli. However, spatial sensing functions independently of cell movement, (indeed we find that even stationary cells can sense chemical gradients), and thus twitching cells using spatial sensing would not be affected by unsteady movement.

5. Twitching cells can, under certain conditions (e.g. when cultured on agar), grow to very high cell densities where they can undergo collective motility [19, 20]. Whilst individual cells can move faster within these collectives compared to solitary cells, a cell's movement in dense collectives is strongly influenced by that of its neighbours, such that individual cells are not in direct control of their movement (e.g., even non-motile cells can be transported by their motile neighbours in collectives; see [19]). Using temporal information to determine the orientation of a chemical gradient in a collective might therefore be challenging because a cell could not discern whether it is moving down a gradient due its own active movement (and thus would benefit from reversing its polarity) or because it is simply being passively shoved by its neighbours in that direction. Thus, in the context of collective motility, spatial sensing – which operates independently from cell movement – could once again offer cells an advantage over temporal sensing.

Taken together, these considerations indicate that planktonic and surface-attached bacteria may be subject to different selection pressures that have led to the evolution of fundamentally different sensing mechanisms for detecting chemical gradients.

References

1. DeLisi C, Marchetti F, Del Grosso G: **A theory of measurement error and its implications for spatial and temporal gradient sensing during chemotaxis**. *Cell Biophys* 1982, **4**(2-3):211-229.

2. Berg HC, Purcell EM: **Physics of chemoreception**. *Biophys J* 1977, **20**(2):193-219.

3. Cai Q, Li Z, Ouyang Q, Luo C, Gordon VD: **Singly flagellated Pseudomonas aeruginosa chemotaxes efficiently by unbiased motor regulation**. *mBio* 2016, **7**(2):e00013.

4. LaBauve AE, Wargo MJ: **Growth and laboratory maintenance of Pseudomonas aeruginosa**. *Curr Protoc Microbiol* 2012, **Chapter 6**:Unit 6E 1.

5. Segall JE, Block SM, Berg HC: **Temporal comparisons in bacterial chemotaxis**. *Proc Natl Acad Sci U S A* 1986, **83**(23):8987-8991.

6. Dusenbery DB: **Spatial sensing of stimulus gradients can be superior to temporal sensing for free-swimming bacteria**. *Biophys J* 1998, **74**(5):2272-2277.

7. Wadhams GH, Armitage JP: **Making sense of it all: bacterial chemotaxis**. *Nat Rev Mol Cell Biol* 2004, **5**(12):1024-1037.

8. Chen YE, Tropini C, Jonas K, Tsokos CG, Huang KC, Laub MT: **Spatial gradient of protein phosphorylation underlies replicative asymmetry in a bacterium**. *Proc Natl Acad Sci U S A* 2011, **108**(3):1052-1057.

9. Tindall MJ, Gaffney EA, Maini PK, Armitage JP: **Theoretical insights into bacterial chemotaxis**. *Wiley Interdiscip Rev Syst Biol Med* 2012, **4**(3):247-259.

10. Jin T: **Gradient sensing during chemotaxis**. *Curr Opin Cell Biol* 2013, **25**(5):532-537.

11. Mika JT, Poolman B: **Macromolecule diffusion and confinement in prokaryotic cells**. *Curr Opin Biotechnol* 2011, **22**(1):117-126.

12. Mullineaux CW, Nenninger A, Ray N, Robinson C: **Diffusion of green fluorescent protein in three cell environments in Escherichia coli**. *J Bacteriol* 2006, **188**(10):3442-3448.

13. Kuhn MJ, Tala L, Inclan YF, Patino R, Pierrat X, Vos I, Al-Mayyah Z, Macmillan H, Negrete J, Jr., Engel JN *et al*: **Mechanotaxis directs Pseudomonas aeruginosa twitching motility**. *Proc Natl Acad Sci U S A* 2021, **118**(30):e2101759118.

14. Kuhn MJ, Macmillan H, Tala L, Inclan Y, Patino R, Pierrat X, Al-Mayyah Z, Engel JN, Persat A: **Two antagonistic response regulators control Pseudomonas aeruginosa polarization during mechanotaxis**. *EMBO J* 2023, **42**(7):e112165.

15. DeLange PA, Collins TL, Pierce GE, Robinson JB: **PilJ localizes to cell poles and is required for type IV pilus extension in Pseudomonas aeruginosa**. *Curr Microbiol* 2007, **55**(5):389-395.

16. Burrows LL: **Pseudomonas aeruginosa twitching motility: type IV pili in action**. *Annu Rev Microbiol* 2012, **66**:493-520.

17. Jin F, Conrad JC, Gibiansky ML, Wong GC: **Bacteria use type-IV pili to slingshot on surfaces**. *Proc Natl Acad Sci U S A* 2011, **108**(31):12617-12622.

18. Block SM, Segall JE, Berg HC: **Impulse responses in bacterial chemotaxis**. *Cell* 1982, **31**(1):215-226.

19. Meacock OJ, Doostmohammadi A, Foster KR, Yeomans JM, Durham WM: **Bacteria solve the problem of crowding by moving slowly** *Nature Physics* 2021, **17**(3):205-210.

20. Gloag ES, Turnbull L, Huang A, Vallotton P, Wang H, Nolan LM, Mililli L, Hunt C, Lu J, Osvath SR *et al*: **Self-organization of bacterial biofilms is facilitated by extracellular DNA**. *Proc Natl Acad Sci U S A* 2013, **110**(28):11541-11546.
