# Supplementary material for: Individual bacterial cells can use spatial sensing of chemical gradients to direct chemotaxis on surfaces
Source: Nat Microbiol. 2024 Sep 2;9(9):2308–22. doi: 10.1038/s41564-024-01729-3 (PMC11371657; doi:10.1038/s41564-024-01729-3)
Supplement: Supplementary file 2 — Reporting Summary [file 41564_2024_1729_MOESM2_ESM.pdf]

nature portfolio

Corresponding author(s): William M. Durham
Kevin R. Foster

Last updated by author(s): 13 June 2024

Reporting Summary

Nature Portfolio wishes to improve the reproducibility of the work that we publish. This form provides structure for consistency and transparency in reporting. For further information on Nature Portfolio policies, see our Editorial Policies and the Editorial Policy Checklist.

Statistics

For all statistical analyses, confirm that the following items are present in the figure legend, table legend, main text, or Methods section.

n/a	Confirmed	
☐	☒	The exact sample size (*n*) for each experimental group/condition, given as a discrete number and unit of measurement
☐	☒	A statement on whether measurements were taken from distinct samples or whether the same sample was measured repeatedly
☐	☒	The statistical test(s) used AND whether they are one- or two-sided<br>*Only common tests should be described solely by name; describe more complex techniques in the Methods section.*
☒	☐	A description of all covariates tested
☒	☐	A description of any assumptions or corrections, such as tests of normality and adjustment for multiple comparisons
☐	☒	A full description of the statistical parameters including central tendency (e.g. means) or other basic estimates (e.g. regression coefficient) AND variation (e.g. standard deviation) or associated estimates of uncertainty (e.g. confidence intervals)
☐	☒	For null hypothesis testing, the test statistic (e.g. *F*, *t*, *r*) with confidence intervals, effect sizes, degrees of freedom and *P* value noted<br>*Give P values as exact values whenever suitable.*
☒	☐	For Bayesian analysis, information on the choice of priors and Markov chain Monte Carlo settings
☒	☐	For hierarchical and complex designs, identification of the appropriate level for tests and full reporting of outcomes
☒	☐	Estimates of effect sizes (e.g. Cohen's *d*, Pearson's *r*), indicating how they were calculated

Our web collection on statistics for biologists contains articles on many of the points above.

Software and code

Policy information about availability of computer code

Data collection	Zen Blue 2012 (Zeiss)<br>NIS-Elements AR v4.51.01 (Nikon)
Data analysis	Commercial software: Matlab 2019b (Mathworks)<br>Open-source software: ImageJ v2.0.0-rc-69/1.52n (https://imagej.net/software/fiji/index) and ImageJ v1.5.4 (https://imagej.net/software/fiji/downloads#Archive - version downloaded from June 02 2014); R, (3.6.1 GUI 1.70 El Capitan build (7684), https://www.r-project.org); The Feature-Assisted Segmenter/Tracker (FAST v2.1, https://mackdurham.group.shef.ac.uk/FAST_DokuWiki/dokuwiki/doku.php?id=start)<br>Custom software: Most of the code used to analyse cell movement in this manuscript has already been described in separate publications (https://doi.org/10.1073/pnas.1600760113 and https://doi.org/10.1371/journal.pcbi.1011524). The code used to generate the findings of this study can be accessed at: https://doi.org/10.15131/shef.data.25800409

For manuscripts utilizing custom algorithms or software that are central to the research but not yet described in published literature, software must be made available to editors and reviewers. We strongly encourage code deposition in a community repository (e.g. GitHub). See the Nature Portfolio guidelines for submitting code & software for further information.

Data

Policy information about availability of data

All manuscripts must include a data availability statement. This statement should provide the following information, where applicable:

- Accession codes, unique identifiers, or web links for publicly available datasets
- A description of any restrictions on data availability
- For clinical datasets or third party data, please ensure that the statement adheres to our policy

Source data for Fig. 2-5 and Extended Data Fig. 1-10 are provided with this paper. Image data (~650 GB) is available from the corresponding authors upon request. All other data that support the findings of this study can be accessed at: https://doi.org/10.15131/shef.data.25800409

Research involving human participants, their data, or biological material

Policy information about studies with human participants or human data. See also policy information about sex, gender (identity/presentation), and sexual orientation and race, ethnicity and racism.

Reporting on sex and gender	Not applicable.
Reporting on race, ethnicity, or other socially relevant groupings	Not applicable.
Population characteristics	Not applicable.
Recruitment	Not applicable.
Ethics oversight	Not applicable.

Note that full information on the approval of the study protocol must also be provided in the manuscript.

Field-specific reporting

Please select the one below that is the best fit for your research. If you are not sure, read the appropriate sections before making your selection.

☒ Life sciences ☐ Behavioural & social sciences ☐ Ecological, evolutionary & environmental sciences

For a reference copy of the document with all sections, see nature.com/documents/nr-reporting-summary-flat.pdf

Life sciences study design

All studies must disclose on these points even when the disclosure is negative.

Sample size	For the manual analyses of repolarisation events (Fig. 4, S8), the number of repolarisation events was determined by the total number of cells present across the sixteen different fields-of-view. For analyses that used automated cell tracking (all other figures), the number of cells that were analysed in each bio-replicate was determined by the cell tracking software.
Data exclusions	No data were excluded from the study.
Replication	Due to the technically challenging nature of microfluidic devices, some attempted experiments did not yield usable data. For example, some experiments ended prematurely because air bubbles inadvertently passed through the microfluidic channels and detached bacteria from the surface. In addition, other experiments had unavoidable imaging artefacts (e.g. shadows cast by parts of the microfluidic system outside the depth of field) that prevented cell segmentation and tracking.<br><br>We note that our automated cell tracking datasets each contain thousands of trajectories enabling within-experiment statistical analyses.
Randomization	Randomization was not relevant in this study because all experiments were performed on bacteria grown from frozen stocks under identical growth conditions.
Blinding	The authors were blind to each other's initial manual classification of intracellular reversals (Fig. 4, S8).

Reporting for specific materials, systems and methods

We require information from authors about some types of materials, experimental systems and methods used in many studies. Here, indicate whether each material, system or method listed is relevant to your study. If you are not sure if a list item applies to your research, read the appropriate section before selecting a response.

Materials & experimental systems

n/a	Involved in the study
☒	☐ Antibodies
☒	☐ Eukaryotic cell lines
☒	☐ Palaeontology and archaeology
☒	☐ Animals and other organisms
☒	☐ Clinical data
☒	☐ Dual use research of concern
☒	☐ Plants

Methods

n/a	Involved in the study
☒	☐ ChIP-seq
☒	☐ Flow cytometry
☒	☐ MRI-based neuroimaging
